# Mangrove taxon mapping from Sentinel-1/2 using a compact one-dimensional network with probability calibration, predictive entropy, and frozen transfer evaluation

**DOI:** 10.3389/fpls.2026.1967679

**Published:** 2026-10-16

**Authors:** Guohao Liu, Shulei Wu, Yaoru Wang, Yihui Zhang, Huandong Chen, Yukai Chen, Zhongqiang Wu, Nan Wang

**Affiliations:** 1 College of Artificial Intelligence, Hainan Normal University, Haikou, China; 2 Key Laboratory of Data Science and Smart Education, Hainan Normal University, Ministry of Education, Haikou, China; 3 Key Laboratory of the Ministry of Education for Coastal and Wetland Ecosystems, College of the Environment and Ecology, Xiamen University, Xiamen, China; 4 College of Life Sciences, Hainan Normal University, Haikou, China

**Keywords:** mangrove taxon mapping, optical and SAR feature fusion, predictive entropy, probability calibration, Sentinel-1, Sentinel-2, spatially grouped cross-validation

## Abstract

**Introduction:**

Mangrove extent maps lack the taxonomic information needed for carbon assessment, restoration evaluation, and management. Taxon mapping at 10 m remains constrained by spectral similarity, mixed pixels, limited reference coverage, and spatial dependence.

**Methods:**

We developed the Inverted-Residual Spectral One-Dimensional Convolutional Neural Network (IR-Spec1D) as a compact classifier for a 14-feature Sentinel-1/2 optical and radar vector. To capture nonlinear optical–radar relationships compactly, inverted-residual and depthwise-separable transformations learn feature interactions, while Efficient Channel Attention emphasizes informative learned channels. Spectral-Domain Dropout reduces dependence on individual inputs through training-time feature masking. The workflow combines this configuration with spatial evaluation, *post hoc* temperature scaling, predictive-entropy analysis, and frozen transfer assessment. The six-category source taxonomy includes the *B. sexangula*/*B. gymnorhiza* complex. Six classifiers were evaluated using April 2024 observations from the selected Dongzhai Harbor study area.

**Results:**

IR-Spec1D achieved the highest mean overall accuracy (0.9720) and macro-F1 (0.9406) under five spatially grouped cross-validation folds. Performance remained comparatively high with a 100-m exclusion buffer. Held-out source-domain diagnostics showed that temperature scaling reduced mean expected calibration error from 0.0346 to 0.0125 without changing categorical predictions. Predictive entropy ranked cross-validated source-domain errors with an AUROC of 0.8808. The frozen ensemble retained an overall accuracy of 0.9450 in both same-site temporal evaluations, whereas four-category closed-set geographic transfer to Gaoqiao yielded 0.7118.

**Discussion:**

The combined evidence characterizes taxon discrimination, held-out probability behavior, and performance retention under specified transfer conditions within a common mangrove-mapping workflow.

## 1 Introduction

Mangrove forests store substantial carbon, reduce coastal exposure to extreme events, and provide habitat and other ecosystem services (Donato et al., 2011; Friess et al., 2019; Menéndez et al., 2020). Extent inventories support area-based monitoring but do not resolve stand composition, which is associated with biomass, productivity, sediment organic carbon, and nutrient properties (Rahman et al., 2024). Taxonomic information also connects remotely sensed observations to taxon-specific biomass relationships and allometric equations used in carbon-stock estimation (Zhu et al., 2015; Hidayatullah et al., 2023; Zhang and Fan, 2025). Restoration outcomes can vary with management strategy and species assemblage (Zhu et al., 2024; Li et al., 2025). Taxon maps therefore complement extent inventories in carbon assessment, restoration evaluation, and monitoring of community composition.

Regional taxon mapping requires detailed discrimination using observations and reference data with sufficient spatial coverage. Field surveys provide direct taxonomic evidence, while unmanned aerial vehicle (UAV) observations and airborne hyperspectral observations resolve fine-scale canopy differences (Lassalle et al., 2023; Yang et al., 2024). Access, acquisition conditions, and survey effort constrain the repeated use of these approaches across extensive intertidal areas. Sentinel-2 provides repeated multispectral coverage and has supported 10 m mapping of multiple mangrove taxa across coastal China (Zhao et al., 2024). Spectral similarity, narrow stands, and mixed canopy–water–substrate pixels nevertheless complicate classification at this resolution (Lassalle et al., 2023; Zhao et al., 2024; Lin et al., 2025). Sentinel-1 synthetic aperture radar (SAR) contributes complementary information about canopy and surface scattering (Fu et al., 2024; Samadzadegan et al., 2025). One-dimensional convolution has been applied to Sentinel-1/2 features for forest-type classification (Bai et al., 2023), while recent mangrove studies combine multisensor observations, ensemble learning, and deep representations (Fu et al., 2023, 2024; Yang et al., 2024; Zhao et al., 2024). These advances leave an unresolved practical question: how should compact multisensor classifiers be configured and evaluated when reliable taxonomic references have limited spatial coverage?

Learning paradigms differ in their supervision, physical constraints, and use of target observations. Examples of supervised or physics-constrained modeling include the meta-learning-based graph convolutional wavelet network for railway subsystem dynamics (Yan et al., 2026); physical relationships also inform radiative-transfer-based Sentinel-2 retrieval (Zérah et al., 2024). Few-shot, weakly/semi-supervised, and prompt-based approaches address limited labeling (Tong et al., 2025; Jia et al., 2025). BCLIP-ADer exemplifies Bayesian prompt-based few-shot anomaly detection (Yang et al., 2026), while prompt adaptation also supports few-shot remote-sensing classification (Zhang et al., 2026). Unsupervised transfer uses unlabeled target observations, as illustrated by spatial–channel graph-interaction transfer for machinery diagnosis (Wang et al., 2026) and domain adaptation for satellite mapping (Mohammadi et al., 2024). The present study uses supervised source-domain learning and evaluates the resulting workflow on temporal and geographic targets without target-domain fitting or adaptation.

Classification accuracy alone does not characterize spatial sensitivity, probability quality, or performance under distribution shift. Spatial dependence can inflate apparent geospatial generalization, while accurate classifiers can still produce poorly calibrated probabilities (Roberts et al., 2017; Valavi et al., 2019; Gawlikowski et al., 2023; Koldasbayeva et al., 2024). Temperature scaling adjusts class probabilities after training (Guo et al., 2017), and recent Earth-observation studies evaluate calibrated confidence alongside spatial, temporal, and cross-sensor generalization (Maslov et al., 2025). We combine spatially explicit source-domain comparisons, held-out evaluation probability diagnostics, and frozen temporal and geographic evaluations.

IR-Spec1D processes a fixed, low-dimensional optical–SAR vector without image-patch neighborhoods. Its established inverted-residual and depthwise-separable operations learn nonlinear representations, Efficient Channel Attention reweights learned channels, and Spectral-Domain Dropout masks individual inputs to regularize feature dependence. The optical and radar inputs form an ordered heterogeneous vector whose prescribed order and fusion design require empirical evaluation. Accordingly, we test global and optical-only rearrangements and compare input-level processing with a modality-specific dual-branch architecture. Spatially grouped comparisons, exclusion buffers, and analyses across seeds and fold assignments assess source-domain classification. Temperature-scaled held-out evaluation probabilities provide calibration diagnostics and predictive entropy, while frozen temporal and geographic experiments assess performance retention.

The study addresses three questions. How does IR-Spec1D compare with machine-learning and deep learning benchmarks under matched spatial validation, and how sensitive is it to its inputs and configuration? How do temperature scaling and predictive entropy characterize source-domain held-out probabilities and classification errors? To what extent is performance retained across acquisition dates and in a prespecified geographic target label space? April 2024 observations from the selected Dongzhai Harbor study area define the source domain. April 2022 and April 2026 observations from the same site and an April 2024 Gaoqiao dataset provide temporal and closed-set geographic evaluations. The contribution combines a compact optical–SAR classifier with explicit assessment of discrimination, probability behavior, spatial sensitivity, and frozen transfer within one mangrove-mapping workflow.

## 2 Materials and methods

### 2.1 Materials

#### 2.1.1 Study areas and experimental domains

Dongzhai Harbor National Nature Reserve is located in northeastern Hainan Province, China (19°51′– 20°01′N, 110°32′–110°37′E; **Figure 1A**). Established in 1980, it was China’s first wetland nature reserve created primarily for mangrove conservation and was designated a Wetland of International Importance in 1992 (National Forestry and Grassland Administration, 2022). Dongzhai Harbor supports one of China’s most taxonomically rich mangrove floras and is a nationally important site for mangrove conservation, restoration, and ecological research (National Forestry and Grassland Administration, 2022). The reserve occupies a drowned river-valley embayment influenced by the Yanzhou and Sanjiang rivers. It contains extensive intertidal mudflats and has a tropical monsoon climate, with mean annual precipitation above 1,600 mm (Zhang and Fan, 2025). Tidal creeks, mudflats, and natural and restored stands form a heterogeneous intertidal landscape. Mangrove community composition and zonation vary with inundation, salinity, substrate, and surface elevation (Krauss et al., 2008; Chen et al., 2023). At 10 m resolution, spectrally similar taxa, fine-scale zonation, narrow stands, and land–water boundaries increase within-scene variability and the occurrence of mixed pixels (Wang et al., 2018; Lassalle et al., 2023; Zhao et al., 2024).

**Figure 1:**
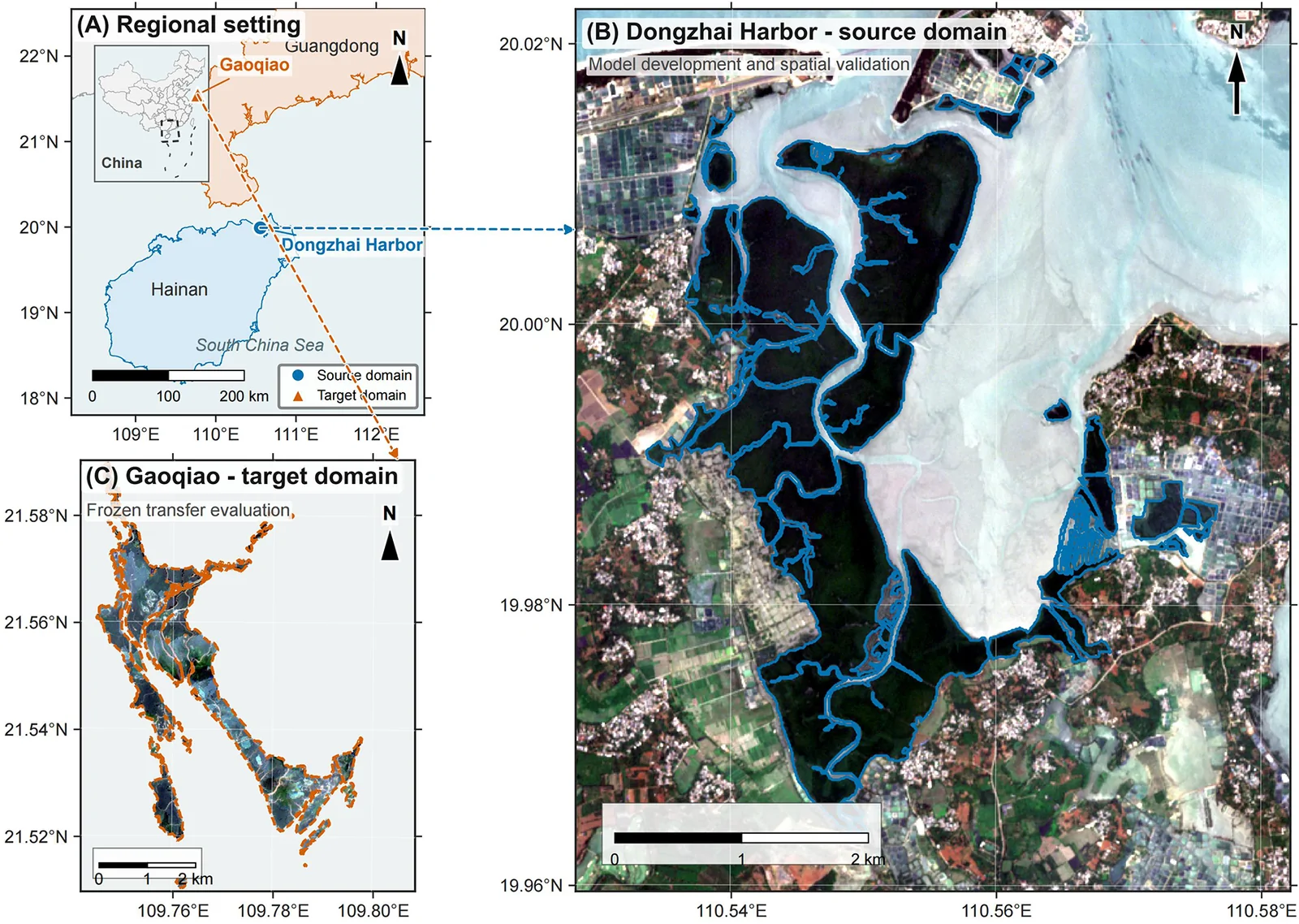
Study areas and experimental domains. **(A)** Regional setting of Dongzhai Harbor in Hainan Province and Gaoqiao in Guangdong Province, China. **(B)** Sentinel-2 true-color composite of the Dongzhai Harbor source domain. **(C)** Sentinel-2 true-color composite of the Gaoqiao target domain. Blue and orange dashed arrows link the regional locations to the detailed panels; corresponding outlines delimit the mangrove analysis extents.

The source-domain analysis focused on a selected mangrove area within Dongzhai Harbor (**Figure 1B**) for which contemporaneous field observations and high-resolution UAV reference imagery were available. UAV coverage over the southern part of the broader Dongzhai Harbor area was constrained by flight restrictions near Haikou Meilan International Airport; consequently, the source-domain reference campaign and taxon-mapping analysis focused primarily on the adequately surveyed area shown in **Figure 1B**. The six categories listed in **Table 1** define the reference-supported canopy taxonomy used for source-domain classification. Field surveys conducted in April 2024 and interpretation of the corresponding UAV orthomosaics provided the taxonomic reference evidence, while paired Sentinel-1/2 observations were used for model development and spatial evaluation.

**Table 1:** Satellite input channels and source-domain reference samples.

Input channels^a^	
Source	Bands or channels
Sentinel-1	VV, VH
Sentinel-2	B1, B2, B3, B4, B5, B6, B7, B8, B8A, B9, B11, B12

Source-domain reference samples^b^	
Classification category	Pixels
*Avicennia marina*	114
*Bruguiera sexangula*/*Bruguiera gymnorhiza* complex	323
*Rhizophora stylosa*	1,241
*Ceriops tagal*	1,088
*Kandelia obovata*	490
*Aegiceras corniculatum*	71
Total	3,327

^a^ Sentinel-1 polarization channels and Sentinel-2 surface-reflectance bands.

^b^ Reference pixels obtained from field surveys and UAV-image interpretation.

Gaoqiao is located in southwestern Guangdong Province, China (21.5100°–21.5900°N, 109.7420°–109.8080°E; **Figure 1**). It forms part of the Zhanjiang Mangrove National Nature Reserve, China’s largest and most spatially concentrated mangrove reserve (Cheng et al., 2025). Natural, planted, and mixed stands support multiple dominant mangrove taxa within a river-influenced intertidal landscape (Gao et al., 2022; Cheng et al., 2025). A previous study in Gaoqiao documented fine-scale variation in local species composition and showed that spatial resolution and combined WorldView-2 and Zhuhai-1 imagery affected species-classification performance (Gao et al., 2022). At 10 m resolution, interspersed taxa with similar canopy responses, together with tidal channels and mudflats, increase mixed pixels and class ambiguity (Gao et al., 2022; Lassalle et al., 2023). Gaoqiao therefore provides an ecologically important and technically challenging geographic target. Field observations and UAV orthomosaics confirmed four categories shared with the Dongzhai Harbor taxonomy, providing the overlap required for closed-set transfer of the frozen source-domain model.

Together, Dongzhai Harbor and Gaoqiao provide ecologically important and complementary study domains within China’s mangrove conservation network. Their pairing was designed to assess frozen model performance within a prespecified shared label space, not to constitute a controlled ecological comparison or establish broad representativeness across Chinese mangrove ecosystems.

#### 2.1.2 Remote-sensing datasets

The source-domain imagery comprised a Sentinel-1 Ground Range Detected (GRD) scene and a Sentinel-2 level-2A scene acquired over Dongzhai Harbor in April 2024. The Sentinel-1 scenes contained vertical-transmit–vertical-receive (VV) and vertical-transmit–horizontal-receive (VH) polarization channels. The temporal target datasets used one scene from each sensor over Dongzhai Harbor in April 2022 and April 2026. The geographic target dataset used one scene from each sensor over Gaoqiao in April 2024.

No multidate compositing was performed. The four datasets retained the same sensor combination and feature order.

Each experimental domain was represented by one Sentinel-2 level-2A acquisition and one Sentinel-1 Interferometric Wide swath GRD acquisition corresponding to the imagery used in the experiments. Exact product identifiers and UTC sensing times are reported in **Supplementary Table S1**. Sentinel-1 observations used VV and VH polarizations, and product-specific incidence-angle statistics were derived from the corresponding SAFE annotation metadata. Sentinel-2 catalog cloud cover and the exact temporal separation between paired Sentinel-1 and Sentinel-2 acquisitions were also recorded. Modeled tidal elevations from TPXO10-atlas-v2 and antecedent rainfall from ERA5 reanalysis were used only to document acquisition conditions and were not included as model predictors.

#### 2.1.3 Reference data and label taxonomy

Source-domain reference data were collected in mid-April 2024 through field surveys and visual interpretation of high-resolution UAV imagery. This combination follows established field-UAV inventory practice in mangrove forests (Otero et al., 2018). Field observations were georeferenced using real-time kinematic positioning, and the dominant mangrove taxon was recorded at each location. Field records and UAV images were used to delineate relatively homogeneous stands with the Region of Interest tool in ENVI 5.6. Reference pixels were then extracted from the coregistered 10 m feature grid. After quality control, the source dataset contained 3,327 pixels distributed among six classification categories (**Table 1**).

Three local case regions were used to examine map behavior in a narrow coastal fringe, an artificial afforestation transect, and a dense mangrove stand. Contemporaneous field observations and UAV orthomosaics provided evidence on taxon occurrence, planting context, and canopy configuration in these regions. Since these observations belonged to the broader source-domain field–UAV inventory, the comparisons were treated as qualitative case analyses rather than an independent accuracy assessment.

*B. sexangula* and *B. gymnorhiza* were difficult to separate in the available observations because of their morphological similarity and frequent cooccurrence. They were therefore represented as a single composite category, the *B. sexangula*/*B. gymnorhiza* complex. This decision aligned the label taxonomy with the resolution of the field and image evidence and avoided unsupported taxon-level assignments.

Although *Sonneratia apetala* occurs within the broader Dongzhai Harbor mangrove system, the finalized 2024 reference inventory for the selected source-domain study area did not contain a sufficiently supported and consistently labeled *S. apetala* reference category established under the same quality-control standard as the six modeled categories. The supervised source-domain taxonomy was therefore restricted to the six reference-supported classification categories listed in **Table 1**. This six-category label space defines the taxa modeled in the present study and should not be interpreted as an exhaustive inventory of all mangrove taxa occurring throughout Dongzhai Harbor.

For temporal transfer, separate reference datasets were compiled for Dongzhai Harbor in April 2022 and April 2026 from contemporaneous field surveys and UAV orthomosaics. Georeferenced sample locations were randomly selected separately for each year and were not required to coincide spatially. Each dataset contained 400 reference samples. In the category order listed in **Table 1**, the 2022 supports were 35, 60, 102, 98, 72, and 33; the 2026 supports were 34, 60, 105, 98, 69, and 34. These samples were used only for numerical evaluation. They did not contribute to model training, configuration decisions, standardization, temperature-scaling parameter estimation, model updating, or map production.

The Gaoqiao dataset contained 288 reference samples from 32 field plots documented by contemporaneous field observations and UAV orthomosaics. Each plot contributed nine adjacent samples, and each of the four shared categories contributed 72 samples. The shared categories were *A. marina*, the *B. sexangula*/*B. gymnorhiza* complex, *R. stylosa*, and *A. corniculatum*. No Gaoqiao reference sample was used for source-domain model development, fine-tuning, or recalibration. Nine samples from one field-observed *Sonneratia apetala* plot were retained separately. These samples were excluded from the four-class closed-set metrics because *S. apetala* was absent from the source taxonomy.

### 2.2 Methods

#### 2.2.1 Workflow overview

The analysis followed a single processing chain from sensor observations to paired classification and predictive-entropy products (**Figure 2**). Sentinel-1 and Sentinel-2 observations were preprocessed, coregistered, and concatenated into a 14-dimensional pixel vector. Reference coordinates defined spatial groups and five cross-validation folds (**Figure 2A**). Each outer iteration used a fixed cyclic allocation: two folds for training, one for internal validation, one for calibration, and one for held-out evaluation. Training observations were used for fitting the StandardScaler and network; internal validation was used only for validation-loss monitoring, early stopping, and checkpoint selection under fixed model settings. The separate calibration fold supported *post hoc* temperature-scaling parameter fitting after the selected checkpoint was restored (**Figure 2C**). For scene-level inference, the fitted scaler, retained checkpoint, and scaling parameter were frozen; temperature-scaled probabilities were averaged across test-time perturbations and then across the five fold-specific models (**Figure 2C**). The final probability vector generated both the maximum-probability category and predictive entropy (**Figure 2D**).

**Figure 2:**
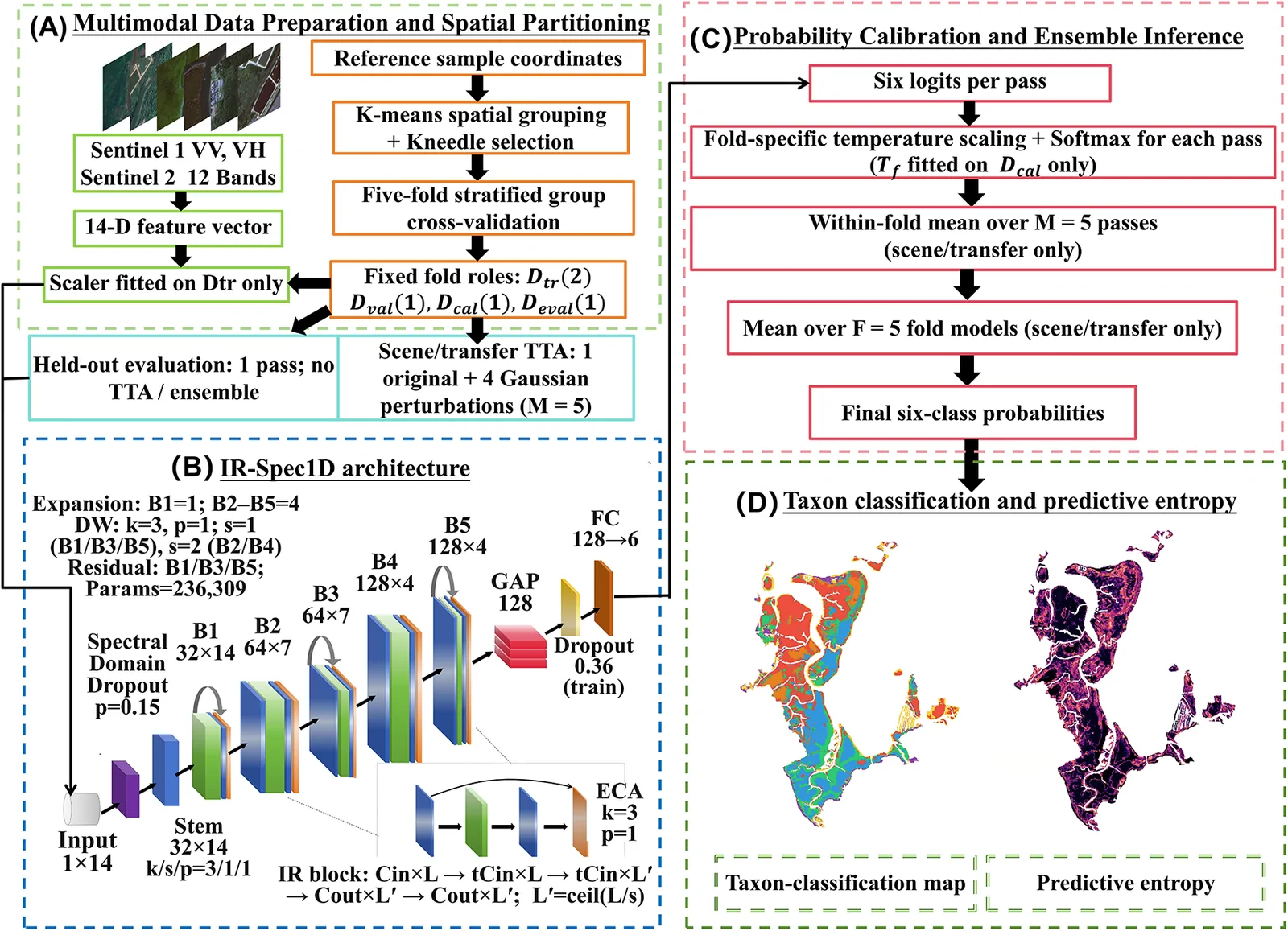
End-to-end IR-Spec1D workflow. **(A)** Multimodal input preparation, spatial grouping, and fold-specific data roles. **(B)** IR-Spec1D feature extraction and classification architecture. **(C)** Fold-specific temperature scaling; within-fold TTA and five-model probability averaging for scene-level and transfer inference. **(D)** Taxon classification and predictive-entropy outputs. Source-domain held-out evaluation uses a single unperturbed forward pass, whereas TTA and five-model probability averaging are restricted to scene-level and transfer inference; dropout is disabled during inference.

#### 2.2.2 Remote-sensing preprocessing and multimodal feature construction

Sentinel-1 Ground Range Detected data were processed in the Sentinel Application Platform (SNAP) 9.0. Processing included orbit-file application and thermal-noise removal, radiometric calibration of sigma nought (*σ*^0^), Refined Lee filtering using a 7 × 7 pixel window, and Range-Doppler terrain correction using the 30-m Shuttle Radar Topography Mission (SRTM) digital elevation model (DEM). Terrain-corrected VV and VH *σ*^0^ backscatter coefficients were converted to decibels. These C-band measurements provide indirect sensitivity to canopy and surface scattering but do not directly measure vertical vegetation structure.

Cloud-affected Sentinel-2 pixels were masked with the Scene Classification Layer (SCL). Bands with a native 10-m grid spacing were retained without resampling. The continuous 20- and 60-m surface-reflectance bands used in the analysis were resampled to 10 m by bilinear interpolation; the categorical SCL was used only as a mask. This operation established a common grid but did not increase the native information content of the coarser bands.

All layers were transformed to World Geodetic System 1984/Universal Transverse Mercator (WGS 84/UTM) zone 49N (EPSG:32649) and aligned to the same 10-m grid. Sentinel-1 and Sentinel-2 coregistration was based on the spatial reference information supplied with the products, followed by manual inspection of corresponding control points. Mangrove masks were established independently for each mapping year and geographic domain rather than by transferring a fixed boundary among datasets. Separate binary masks were prepared for Dongzhai Harbor in 2022, 2024, and 2026 and for Gaoqiao in 2024. For each dataset, a preliminary mangrove extent was derived from the corresponding imagery and subsequently checked and refined using available high-resolution reference imagery, UAV imagery where available, field information, and manual visual interpretation. The final binary mask was used only to define the spatial domain for taxon inference and did not provide taxonomic labels or predictors to the classifier.

To assess the influence of plausible mask-boundary uncertainty on the taxon-classification maps, each final mapping footprint was eroded inward by nominal distances of 10 and 20 m (one and two 10-m pixels, respectively). Existing map outputs were then restricted to the retained interior pixels, and taxon proportions were recalculated using only pixels with valid taxon predictions. No model retraining, recalibration, or target-domain updating was performed. This analysis was treated as a taxon-map boundary robustness check rather than as an independent accuracy assessment of mangrove extent.

For each valid mangrove pixel *r*, the Sentinel-2 level-2A surface-reflectance component was defined as

(1) $$x_{r,\text{S}2}={\left[x_{r,\text{B}1},x_{r,\text{B}2},x_{r,\text{B}3},x_{r,\text{B}4},x_{r,\text{B}5},x_{r,\text{B}6},x_{r,\text{B}7},x_{r,\text{B}8},x_{r,\text{B}8\text{A}},x_{r,\text{B}9},x_{r,\text{B}11},x_{r,\text{B}12}\right]}^{T}\in {\mathbb{R}}^{12}.$$

The 12 optical features were then concatenated with Sentinel-1 VV and VH backscatter to form the complete multimodal feature vector in Equation 2

(2) $$x_{r}={\left[x_{r,\text{S}2}^{T},{\text{x}}_{r,\text{VV}},{\text{x}}_{r,\text{VH}}\right]}^{T}\in {\mathbb{R}}^{14}.$$

The optical component spans the visible, red-edge, near-infrared, and shortwave-infrared regions. Sentinel-2 and Sentinel-1 were therefore fused at the input-feature level before fold-specific standardization. The feature order in Equations 1, 2 was fixed across the source and target datasets. The resulting input is treated as an ordered heterogeneous multimodal feature vector, not as a physically continuous spectrum: adjacency along the 14-position axis encodes a prescribed representation rather than uniform spectral proximity across the optical–radar boundary. Sensitivity to this prescribed order and to input-level versus modality-specific feature extraction was evaluated directly as described below.

#### 2.2.3 Spatial grouping, data partitioning, and fold-specific standardization

**Figure 3A** summarizes the workflow from candidate-specific K-means clustering and group-disjoint proxy validation to the final *K* = 80 five outer partition folds. Reference coordinates in WGS 84/UTM zone 49N were partitioned using K-means so that spatial groups, rather than individual pixels, could be assigned to held-out evaluation folds. Candidate values were *K* ∈ {10,20,30,50,80,120,200}, with random seed 42 and 10 centroid initializations. For each candidate, a single *K*-specific, group-disjoint training–validation holdout was assigned, with approximately 30% of the spatial groups assigned to validation. From 2,000 candidate group splits, the procedure retained those containing all six categories in both partitions and selected the split minimizing the combined class-proportion and validation-size deviations. Model performance was not used to select the split. The spatial groups were recomputed for each value of *K*; therefore, validation membership could differ among candidates.

**Figure 3:**
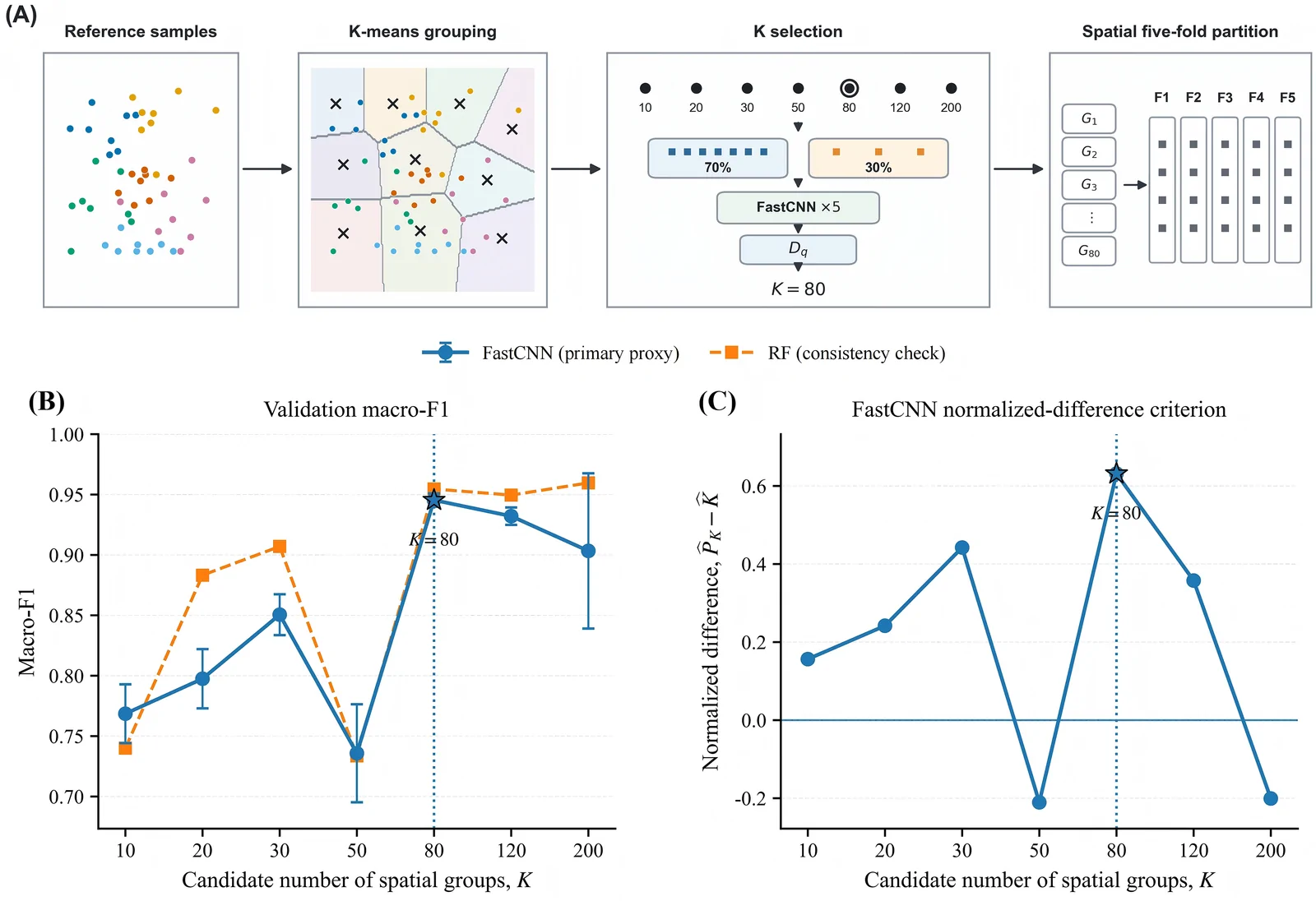
Spatial-group selection and outer-fold construction. **(A)** Candidate-specific K-means grouping and group-disjoint proxy validation select *K* = 80, followed by intact-group assignment to five outer folds. Colored points denote categories; shaded polygons and gray squares denote spatial groups. **(B)** Validation macro-F1 for FastCNN and the fixed-seed RF consistency check. FastCNN values show the mean, and error bars show standard deviation across five initialization seeds; RF was evaluated once per candidate and has no error bars. **(C)** The normalized-difference criterion, *D_q_* = *P_q_* − *K_q_*, reached its maximum at *K* = 80. Candidate values are equally spaced on both horizontal axes.

A StandardScaler was fitted only on the training partition of each candidate holdout. A fixed lightweight one-dimensional convolutional neural network, denoted FastCNN in **Figure 3**, served only as the selection proxy. It comprised a 1D convolution with 16 output channels (kernel size of three, padding of one), ReLU activation, MaxPool1D with kernel size of two, a second 1D convolution with 32 output channels (kernel size of three, padding of one), ReLU activation, adaptive global average pooling, flattening, and a linear classification layer. The proxy was trained for 30 epochs with Adam at a learning rate of 1 × 10^−3^, and training was repeated with five fixed initialization seeds. Let *P_q_* denote the mean validation macro-averaged F1 (macro-F1) for candidate *q*. A Kneedle-inspired normalized-difference criterion (Satopaa et al., 2011) was defined by Equation 3

(3) $$D_{q}={\hat{P}}_{q}-{\hat{K}}_{q},$$

where ${\hat{P}}_{q}$ and ${\hat{K}}_{q}$ are the mean validation macro-F1 and candidate group count after separate min–max normalization to [0,1]. The selected operating point was $K^{*}=K_{q^{*}}$, where $q^{*}=\arg\max_{q}D_{q}$. A fixed-seed random forest (RF) was evaluated on the same candidate-specific holdouts only as an auxiliary model consistency check. It used RandomForestClassifier(random_state=42, n_jobs=-1), with all other settings at scikit-learn defaults, and did not participate in the selection of K^*^. Consistency was supported when the RF macro-F1 at K^*^ was within 0.01 of its maximum across the tested candidates. This procedure selected *K* = 80.

The value *K* = 80 was selected once using proxy-validation results from the complete source-domain reference dataset. It was then fixed for all formal model comparisons, ablations, calibration analyses, and source-domain performance summaries, without being reselected within each outer iteration. The reported source-domain estimates are therefore conditional on this data-informed grouping configuration and may retain optimism related to selection.

The 80 groups were assigned to *F* = 5 outer folds using StratifiedGroupKFold(n_splits=5, shuffle=False), without a random-state argument. The grouping seed of 42 and 10 initializations were applied to K-means, not to this outer-fold assignment. Each outer iteration used intact folds in a fixed cyclic four-role allocation. Fold *f* formed $D_{f}^{\text{eval}}$; the next fold formed $D_{f}^{\text{val}}$, the following fold formed $D_{f}^{\text{cal}}$, and the remaining two folds formed $D_{f}^{\text{tr}}$. Fold indices wrapped cyclically from five to one; **Algorithm S1** provides the complete mapping. This allocation specifies fold counts rather than equal sample proportions. The two training folds supplied all observations used to fit the fold-specific StandardScaler and network parameters. Under fixed model settings, $D_{f}^{\text{val}}$ was used only for validation-loss monitoring, early stopping, and checkpoint selection, not for model-hyperparameter search or optimization. After the selected checkpoint was restored, the separate calibration fold supplied logits and labels solely for fitting the temperature-scaling parameter. After *K* had been fixed, held-out evaluation observations were excluded from standardization, model fitting, early stopping, checkpoint selection, configuration changes, and temperature-scaling parameter estimation. The fitted scaler, retained checkpoint, and temperature-scaling parameter were subsequently frozen for scene-level, temporal-transfer, and geographic-transfer inference.

For each fold model, a StandardScaler was fitted only to $D_{f}^{\text{tr}}$. If ***μ****_f_* and ***s****_f_* denote the resulting feature means and scales, an input vector was transformed using Equation 4:

(4) $${\tilde{x}}_{r,f}=\left(x_{r}-\mu_{f}\right)⊘s_{f},\;$$

where ⊘ denotes element-wise division. The fitted *µ_f_* and **s***_f_* were applied without refitting to the internal validation, calibration, and held-out evaluation subsets and to all scene-level and transfer inference data processed by fold model *f*.

Each spatial group was kept intact during outer-fold assignment so that it could not contribute observations to both model development and held-out evaluation (Roberts et al., 2017; Valavi et al., 2019). All six categories were represented in every held-out evaluation fold; *A. corniculatum* was the smallest source category, with 71 pixels. K-means partitioning used random seed 42.

After the outer-fold assignments had been fixed, we performed a spatial audit at the reference-region of interest (ROI), pixel/group, and geographic-separation levels. Manual checking against the original field/UAV-informed reference ROI delineations confirmed that none of the 373 original ROIs was split across outer folds (373/373 intact; 0 split). The integrity check retained these original ROI units. All 80 coordinate-defined spatial groups were likewise preserved during outer-fold assignment (0/80 split), and no duplicated reference-pixel coordinates occurred across folds. For each held-out evaluation reference pixel, Euclidean distance to the nearest training observation was calculated using the projected coordinates. Spatial clustering of pooled cross-validated source-domain classification errors was quantified using global Moran’s *I* for a binary error indicator (1 = misclassified, 0 = correct). The primary spatial weights were row-standardized eight-nearest-neighbor weights, with significance assessed by 9,999 permutations; four- and 12-nearest-neighbor and 20-, 30-, and 50-m distance-band definitions were retained as supplementary sensitivity checks.

To directly evaluate robustness to short-range proximity between training and evaluation observations, we additionally conducted buffered spatial-separation analyses of the final IR-Spec1D configuration. For each fixed outer evaluation fold, training observations within 20, 50, or 100 m of any held-out evaluation observation were excluded before model fitting, while the held-out evaluation observations were unchanged. These buffered analyses complement the primary spatially grouped benchmark in **Table 2** and follow remote-sensing practice emphasizing intact spatial units and explicit geographic separation during validation (Meng et al., 2026; Zhan et al., 2026; Orndahl et al., 2025; Rodda et al., 2024). At the representative 50-m buffer, *k*-nearest neighbors (KNN), RF, and 1D-ConvNeXt were evaluated using the same retained training observations, held-out evaluation folds, preprocessing, and fold-specific data roles as IR-Spec1D.

**Table 2:** Performance of IR-Spec1D and five benchmark models under matched fivefold spatially grouped cross-validation with *K* = 80.

Model	OA	Macro-F1
SVM	0.8819 ± 0.0407	0.8091 ± 0.0917
KNN	0.9601 ± 0.0254	0.8671 ± 0.0906
RF	0.9423 ± 0.0158	0.8730 ± 0.0661
1D-ConvNeXt	0.9577 ± 0.0253	0.8698 ± 0.1098
Lightweight 1D-ViT	0.9418 ± 0.0340	0.8652 ± 0.0746
**IR-Spec1D**	**0**.**9720** ± **0**.**0188**	**0**.**9406** ± **0**.**0456**

Values are mean ± standard deviation across the five held-out evaluation folds. The standard deviation denotes fold-to-fold variation, not repeated-initialization variation. Bold values identify the highest mean overall accuracy and highest mean macro-F1, respectively, among the six compared classifiers. They do not denote a statistical significance test.

#### 2.2.4 IR-Spec1D architecture and model optimization

IR-Spec1D transforms the standardized 14-feature vector in Equation 4 into six source-domain class logits. A one-dimensional stem convolution with kernel size three maps the input to 32 channels and is followed by batch normalization and a sigmoid linear unit (SiLU) activation. Five inverted residual blocks then use output-channel dimensions of 32, 64, 64, 128, and 128; their expansion ratios are 1, 4, 4, 4, and 4, respectively (**Figure 2B**; complete tensor shapes and parameter counts are given in **Supplementary Table S11A**). **Supplementary Table S11B** defines the notation used in the model and inference equations, and **Supplementary Algorithm S1** gives the complete fold-wise training, validation-loss monitoring, early stopping, checkpoint selection, separate calibration-set temperature-scaling parameter fitting, held-out source-domain evaluation, and frozen-inference procedure.

The one-dimensional operations provide parameter-efficient nonlinear interactions along the fixed feature axis, followed by channel reweighting and global aggregation. This is a modeling choice for a compact, low-dimensional heterogeneous vector, not a claim that Sentinel-2 reflectance and Sentinel-1 backscatter form a physically continuous spectrum. The task-specific rationale is that the model operates on a fixed, low-dimensional, coregistered optical–SAR feature vector in a reference-limited setting, using compact nonlinear transformations and channel reweighting without requiring spatial image patches or a substantially larger feature extractor. In particular, local convolutional neighborhoods that cross the optical–radar boundary have no uniform wavelength interpretation. We therefore evaluate the dependence of the learned representation on the prescribed global and optical-band orderings and compare it with an approximately parameter-matched modality-specific dual-branch alternative. These empirical controls constrain the architectural interpretation; they do not establish that any one feature ordering or fusion design is physically optimal.

For an intermediate tensor **h** with matched input and output dimensions, a simplified inverted residual mapping is given by Equation 5:

(5) $$ℛ\left(h\right)=h+{\text{Conv}}_{1\times 1}^{\text{proj}}\left[{\text{DW}\text{Conv}}_{k=3}\left({\text{Conv}}_{1\times 1}^{\text{exp}}\left(h\right)\right)\right],$$

where the pointwise expansion convolution increases the intermediate channel dimension, the depthwise convolution operates separately along each channel, and the pointwise projection convolution returns the representation to the required output dimension. The expanded representation limits premature information compression (Sandler et al., 2018), whereas depthwise separable convolution reduces the parameter and arithmetic requirements relative to a standard convolution with matched dimensions (Zhang et al., 2021).

Efficient Channel Attention (ECA) models local cross-channel interactions without an explicit dimensionality-reduction layer (Wang et al., 2020). Global average pooling (GAP) summarizes an intermediate tensor **H** before the attention weights are calculated using Equation 6:

(6) $$\omega =\sigma \left[{\text{Conv}1\text{D}}_{k=3}\left(\text{GAP }(H)\right)\right],$$

where *σ* is the sigmoid function, and ω reweights **H** by channel-wise multiplication. Let $H_{r,f}^{\text{out}}$ denote the final ECA-reweighted feature tensor for pixel *r* under fold model *f*. The classification head produces the six class logits as follows (Equation 7)

(7) $$z_{r,f}=W_{f}\;D\left[\text{GAP}\left(H_{r,f}^{\text{out}}\right)\right]+b_{f},\;z_{r,f}\in {\mathbb{R}}^{C},\;C=6,$$

where **W***_f_* and **b***_f_* are the parameters of the fully connected layer, and *D* denotes Dropout. The vector $z_{r,f}=\left[z_{r,f,1},\ldots ,z_{r,f,6}\right]$ contains the six unnormalized category scores, or logits, rather than class probabilities. The classification head does not apply Softmax. During training, these logits enter the class-weighted cross-entropy loss. For probability calibration and inference, the same logits are divided by the fold-specific temperature-scaling parameter *T_f_* before Softmax, as defined by the calibration procedure in Equations 9a, 9b and the pass-specific transformation in Equation 11b. Dropout was enabled only during model training and was disabled during internal-validation loss monitoring, calibration-set temperature-scaling parameter fitting and held-out evaluation, and scene-level or transfer inference.

In addition to conventional activation dropout (Srivastava et al., 2014), IR-Spec1D applies Spectral-Domain Dropout to the complete set of input features with probability 0.15. This Bernoulli feature mask was motivated by channel- and modality-dropout methods that reduce dependence on a single signal source (Neverova et al., 2016; Lapkovskis et al., 2025; Zhou et al., 2026). It was used as a training regularizer, not as a physical simulation of cloud contamination, SAR speckle, atmospheric-correction errors, missing acquisitions, or sensor failure.

Class imbalance was addressed with weighted cross-entropy. Within fold (*f*), the weight for class *c* was calculated as Equation 8:

(8) $$w_{f,c}=\frac{N_{f}^{tr}}{CN_{f,c}^{tr}},\;C=6,$$

where $N_{f}^{\text{tr}}$ is the number of training samples and $N_{f,c}^{\text{tr}}$ is the corresponding class support. A label-smoothing coefficient of 0.01 regularized the training targets (Müller et al., 2019). Independent Gaussian noise with a standard deviation of 0.05 was added after standardization during training.

AdamW optimization used an initial learning rate of 8 × 10^−4^ and weight decay of 1 × 10^−3^ (Loshchilov and Hutter, 2019). A cosine-annealing schedule reduced the learning rate during training (Loshchilov and Hutter, 2017). Each fold model was trained for at most 200 epochs with a batch size of 64. Early stopping monitored loss on the internal validation subset $D_{f}^{\text{val}}$ with a patience of 25 epochs and restored the weights from the epoch with the lowest validation loss. The primary IR-Spec1D models reported in the formal benchmark used training seed 42, and scene-level inference used a batch size of 4,096.

#### 2.2.5 Fold-specific probability calibration and ensemble inference

Softmax probabilities obtained after cross-entropy training need not correspond to empirical correctness frequencies (Guo et al., 2017; Gawlikowski et al., 2023). After network training and checkpoint selection using the internal validation subset, a positive temperature-scaling parameter was fitted for each fold model using only the logits and labels of its separate calibration subset $D_{f}^{\text{cal}}$. Evaluating the classification head in Equation 7 for calibration sample *n* produced the logit vector $z_{n,f}^{\text{cal}}$. Dividing this vector by a candidate scaling factor *T* and applying Softmax yielded the probabilities used in the regularized negative-log-likelihood objective. The fitted parameter was obtained by the minimization in Equation 9a using the objective in Equation 9b:

(9a) Tf=argmin T>0ℒcal,f(T),

(9b) ℒcal,f(T)=−1Nfcal∑n=1Nfcal∑c=1C yn,clog[Softmaxc(zn,fcalT)]+λ(logT)2,

where $N_{f}^{\text{cal}}$ is the calibration-subset size and *y_n,c_* is the one-hot reference label. The temperature-scaling parameter is a positive scalar applied to model logits. The regularization coefficient in Equation 9b was *λ* = 1 × 10^−3^, and the minimization in Equation 9a was solved with the limited-memory Broyden–Fletcher–Goldfarb–Shanno (L-BFGS) algorithm. The fitted *T_f_* was fixed before held-out evaluation and subsequently retained unchanged for scene-level and transfer inference. Applying the same positive scalar to every logit preserves the maximum-logit category; *T_f_* *>* 1 reduces probability concentration, whereas *T_f_* < 1 increases it.

Scene-level and transfer inference used perturbation-based test-time augmentation (TTA) (Wang et al., 2019). For pixel *r* and fold model *f*, the standardized vector from Equation 4 generated the *M* = 5 inference inputs defined in Equations 10a–c:

(10a) $$x_{r,f}^{\left(m\right)}={\tilde{x}}_{r,f}+\xi_{r,f}^{\left(m\right)},$$

(10b) $$\xi_{r,f}^{\left(1\right)}=0,$$

(10c) $$\xi_{r,f}^{\left(m\right)}∼N\left(0,0.05^{2}I_{14}\right),\;m=2,\ldots ,M.$$

Both activation dropout and Spectral-Domain Dropout were disabled at this stage; TTA represented input perturbation rather than Monte Carlo dropout. TTA averaged predictions over a local neighborhood of the standardized observed feature vector and is used for local probability stabilization, not as a physical sensor-degradation model. Combining predictions across test-time augmentations follows the general TTA principle (Picek et al., 2022), while small Gaussian input perturbations have also been used for model-sensitivity analysis in plant deep-learning applications (Patra and Sahoo, 2024). Let $g_{\theta_{f}}$ denote the complete trained fold model, including the classification head in Equation 7. Each TTA input produced the pass-specific logit vector $z_{r,f}^{\left(m\right)}$ in Equation 11a. Equation 11b then divided these logits by the fitted temperature-scaling parameter *T_f_* and applied Softmax to obtain the corresponding temperature-scaled class probabilities:

(11a) $$z_{r,f}^{\left(m\right)}=g_{\theta_{f}}\left(x_{r,f}^{\left(m\right)}\right),$$

(11b) $$\pi_{r,f,c}^{\left(m\right)}=\frac{\exp\left(z_{r,f,c}^{\left(m\right)}/T_{f}\right)}{\sum_{j=1}^{C}\exp\left(z_{r,f,j}^{\left(m\right)}/T_{f}\right)}.$$

Temperature scaling and Softmax were thus applied before any averaging. Equation 12a first averaged the pass-level probabilities within each fold, and Equation 12b then averaged the resulting probabilities across the five fold models:

(12a) $$q_{r,f,c}=\frac{1}{M}\sum_{m=1}^{M}\pi_{r,f,c}^{\left(m\right)},$$

(12b) $${\bar{\pi }}_{r,c}=\frac{1}{F}\sum_{f=1}^{F}q_{r,f,c},\;M=F=5.$$

Equations 12a, b define probability-level averaging; model weights were not averaged. Each pass-level vector is a Softmax distribution $\sum_{c=1}^{C}{\bar{\pi }}_{r,c}=1$, except for differences attributable to numerical precision.

#### 2.2.6 Classification-category and predictive-entropy mapping

The final class-probability vector in Equation 12b produced two complementary outputs:

(13a) y^r=argmax cπ¯r,c,

(13b) $$H_{r}=-\sum_{c=1}^{C}{\bar{\pi }}_{r,c}\text{ log }\left({\bar{\pi }}_{r,c}+\in_{\text{num}}\right).$$

Here, ${\hat{y}}_{r}$ in Equation 13a is the classification category, and *H_r_* in Equation 13b is the predictive entropy. The natural logarithm was used, so entropy is expressed in nats; *∈*_num_ prevents evaluation of log(0). Higher entropy indicates that probability is distributed more evenly among competing categories and therefore denotes greater model ambiguity (Loosvelt et al., 2012; Kendall and Gal, 2017). The classification and entropy values were projected onto the common image grid to form the paired products shown in **Figure 2D**.

Predictive entropy was used as an exploratory cue for inspection prioritization, not as a substitute for independent validation (McRoberts, 2011). A high value was not assumed to identify a classification error, a mixed stand, an ecological boundary, or a taxon absent from the training taxonomy.

Predictive-entropy patterns were quantitatively evaluated using the fixed cross-validated source-domain predictions (*n* = 3,327) and the formal fold-specific temperature scaling. Predictive entropy was calculated from probabilities pooled across the held-out evaluation sets $D_{f}^{\text{eval}}$, using checkpoints selected on $D_{f}^{\text{val}}$ and temperature-scaling parameters fitted only on $D_{f}^{\text{cal}}$. Classification-error association was quantified by comparing entropy between correct and misclassified observations and by evaluating entropy as an error-ranking score using the area under the receiver operating characteristic curve (AUROC) and the area under the precision–recall curve (AUPRC). Distance to the nearest mangrove/non-mangrove interface was calculated in meters from the final 2024 binary mangrove mask in projected coordinates; raster-image edges were excluded from the boundary definition. Boundary-distance classes were prespecified as 0–20, 20–50, and *>* 50 m. Local Sentinel-2 spectral heterogeneity was calculated in a fixed 3 × 3-pixel neighborhood centered on each reference location. For each of the 12 Sentinel-2 bands listed in **Table 1**, the local standard deviation was calculated when at least six of nine pixels were valid; the band-specific standard deviations were then z-standardized across valid cross-validated observations and averaged, requiring at least 10 of 12 valid bands. Uncertainty intervals were estimated using 5,000 cluster-bootstrap replicates in which the 80 fixed spatial blocks were resampled with replacement and all observations belonging to a selected block were retained.

For the three local case regions shown in **Figure 4**, taxonomic reference interpretations were established independently of the model outputs through repeated field surveys of the focal plots by the research team and visual interpretation of high-resolution orthophotography. The same georeferenced focal boxes were then used across the classifier outputs, predictive-entropy maps, and orthophoto panels. Region 1 was interpreted as an *R. stylosa*-dominated stand with a mixed canopy; Region 2 as a mixed planting strip in which the outlined focal plot was dominated by *A. corniculatum*, with *R. stylosa* and *A. marina* also present; and Region 3 as an *R. stylosa* core with possible *C. tagal* occurrence toward the margins. These field/orthophoto references were used for local case interpretation only and were not used for model fitting, probability calibration, or quantitative accuracy estimation.

**Figure 4:**
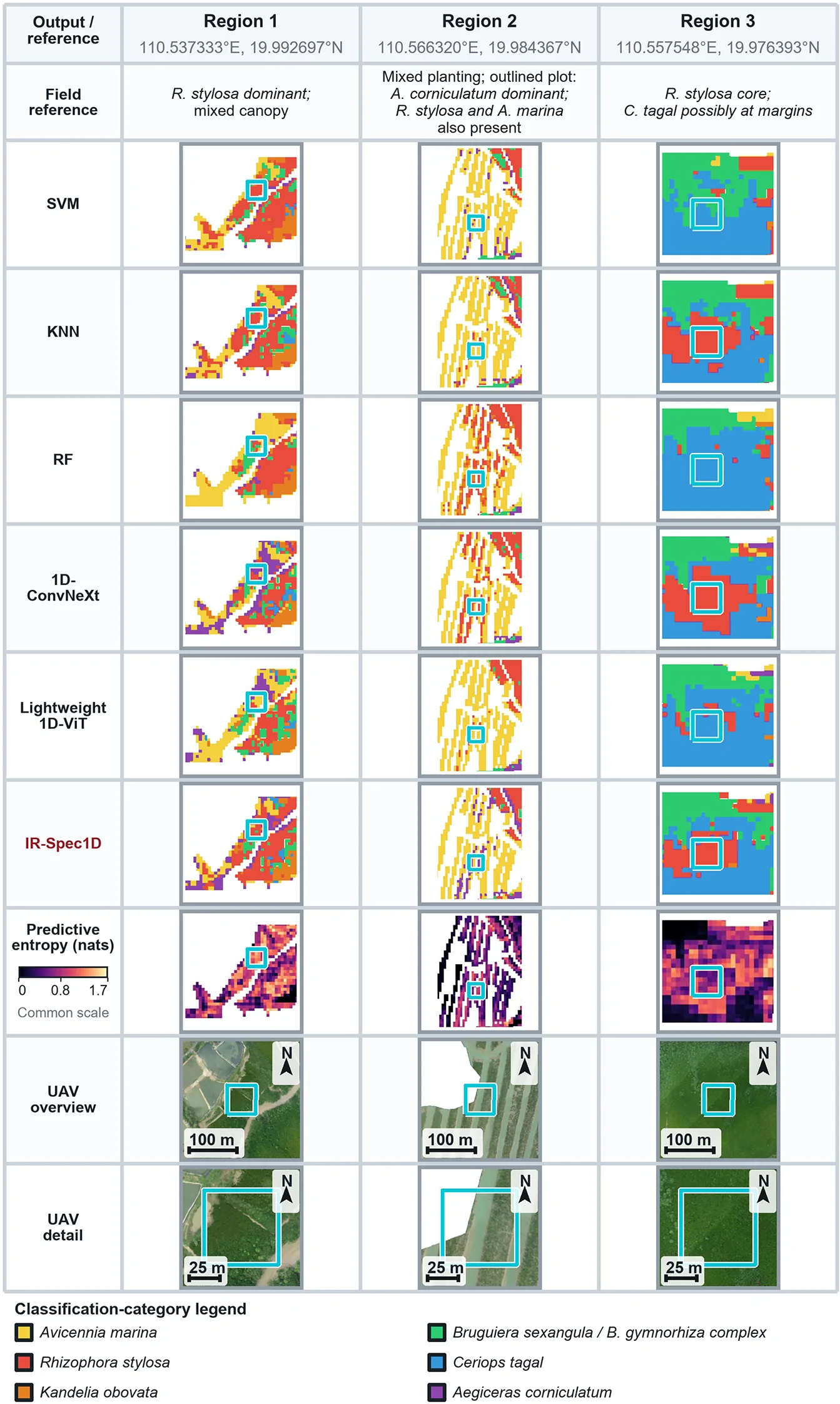
Local taxon predictions and predictive entropy. The field/UAV reference row provides interpretations for three representative Dongzhai Harbor case regions established independently of model outputs. Region 1 is an *R. stylosa*-dominated mixed canopy. Region 2 is a mixed planting strip; the outlined focal plot is dominated by *A. corniculatum*, with *R. stylosa* and *A. marina* also present. Region 3 has an *R. stylosa* core, with possible *C. tagal* at the margins. Cyan boxes identify the same georeferenced focal area across classifier outputs, entropy maps, and orthophoto panels. These references provide qualitative ecological context, not additional samples for model fitting or numerical accuracy estimation. Entropy panels share a common color scale in nats.

### 2.3 Experimental setup and evaluation protocols

#### 2.3.1 Computational environment and implementation settings

Experiments completed before the revision were conducted on a Windows 10 Professional workstation equipped with an NVIDIA GeForce RTX 4090 graphics processing unit (GPU) and 32 GB RAM. The original software stack comprised Python 3.10, PyTorch 2.0.1 with Compute Unified Device Architecture (CUDA) 11.8, scikit-learn 1.3.0, and NumPy 1.24.3 in a 64-bit Conda environment. Additional revision-stage sensitivity analyses and computational profiling were conducted on a Windows 11 workstation with an Intel Core i7-13700KF central processing unit (CPU) and an NVIDIA GeForce RTX 4090 GPU using Python 3.13.5, PyTorch 2.10.0+cu126, CUDA 12.6, and CUDA Deep Neural Network library (cuDNN) 9.10.2. Unless otherwise specified, revision-stage computations used a random seed of 42; deterministic cuDNN settings were used for neural-model runs. Computational profiling details are reported in Section 2.3.7 and **Supplementary Tables S12**, **S13**.

No model-hyperparameter search or optimization was conducted before or during the formal benchmark using five folds. Each classifier used a single configuration fixed before formal evaluation and applied unchanged across all five outer folds. For neural models, the internal-validation subset D*_f_*^val^ was used only for validation-loss monitoring, early stopping, and checkpoint selection; it was not used to select the network architecture, learning rate, weight decay, dropout probability, batch size, or other model hyperparameters. Complete model configurations are reported in **Supplementary Table S3**. The *K*-selection procedure described in Section 2.2.3 concerns spatial-validation design rather than model-hyperparameter tuning.

#### 2.3.2 Source-domain metrics and fold-level aggregation

Source-domain discrimination was summarized using overall accuracy (OA), macro-F1 score, and category-level user’s accuracy (precision), producer’s accuracy (recall), and F1 score. expected calibration error (ECE) and negative log-likelihood (NLL) provided fold-wise *post hoc* calibration diagnostics. Each sample contributed to the source-domain metrics only when its spatial group formed the held-out evaluation fold $D_{f}^{\text{eval}}$, using the scaler fitted on $D_{f}^{tr}$, the checkpoint selected on $D_{f}^{\text{val}}$, and the temperature-scaling parameter fitted on $D_{f}^{\text{cal}}$. These predictions used a single unperturbed forward pass. TTA and the five-model ensemble in Equation 12b were restricted to scene-level and transfer inference.

ECE was calculated using Equation 14 with 15 equal-width confidence intervals that divided [0,1]. For fold (*f*), the mean confidence and empirical accuracy were calculated in each nonempty interval *B_f,b_*, and their absolute difference was weighted by the interval’s fraction of the held-out evaluation fold, whose sample count is $N_{f}^{\text{eval}}$:

(14) $${\text{ECE}}_{f}=\sum_{b=1}^{15}\frac{\left|B_{f,b}\right|}{N_{f}^{\text{eval}}}\left|\text{acc }(B_{f,b})-\text{conf }(B_{f,b})\right|.$$

NLL for the same held-out evaluation fold was calculated using Equation 15:

(15) $${\text{NLL}}_{f}=-\frac{1}{N_{f}^{\text{eval}}}\sum_{n=1}^{N_{f}^{\text{eval}}}\log\left(\pi_{n,f,y_{n}}\right),$$

where *π_n,f,yn_* is the probability assigned by fold model *f* to the reference category of sample *n*. Equations 14, 15 were applied to the probabilities before and after temperature scaling. The temperature-scaling parameter was fitted only on $D_{f}^{\text{cal}}$; these diagnostics were computed on $D_{f}^{\text{eval}}$, which was not used for checkpoint selection or temperature-scaling parameter fitting. Final OA, macro-F1, ECE, and NLL were summarized by their arithmetic means across the five held-out evaluation folds. Reported standard deviations, including those in **Table 2**, represent fold-to-fold variation rather than repeated-initialization variation.

#### 2.3.3 Benchmark models and matched comparison protocol

IR-Spec1D was compared with five benchmark models: a support vector machine (SVM), KNN, RF, one-dimensional ConvNeXt (1D-ConvNeXt), and a lightweight one-dimensional vision transformer (1D-ViT). All six models used the same 14 input features, source-domain categories, precomputed spatial groups, outer-fold assignments, fold-specific preprocessing, cyclic 2/1/1/1 data-role assignments, and evaluation metrics. Where stochastic initialization or estimator randomness was applied in the primary formal benchmark, the corresponding fixed seed was set to 42. Consistent with the fixed-configuration protocol, the neural models used the same maximum epoch budget, batch size, and early-stopping rule. For full-scene mapping, each benchmark was retrained using the same two-fold training assignment in each outer iteration. Neural early stopping and checkpoint selection used the assigned internal-validation fold. The resulting fold-specific predictors were used jointly to produce a single full-scene map per benchmark rather than selecting the output from a single fold.

#### 2.3.4 Component ablation and simulated feature omission

Leave-one-component-out ablations used the same cyclic 2/1/1/1 fold-role allocation, preprocessing, optimization framework, and evaluation metrics as the complete IR-Spec1D model. Each ablation removed one component or sensor input group while retaining the remainder of the configuration. The resulting changes quantify the contribution of a component under this matched design; they do not establish a unique causal mechanism.

Sensitivity to simulated random feature omission was evaluated separately. All 14 features were eligible, and omission counts ranged from 0 to 10. At each omission level and within each fold, five feature subsets were sampled without replacement. Every omitted feature was replaced by its mean calculated exclusively from the corresponding training subset. Metrics were first averaged over the five repetitions within each fold. The plotted curves report the mean of these fold-level values, and the shaded bands denote plus or minus one standard deviation across folds. This controlled perturbation does not reproduce the physical effects of cloud contamination or sensor failure.

#### 2.3.5 Input-order and modality-fusion sensitivity

To examine sensitivity to the prescribed order of the heterogeneous inputs, we first used three matched global arrangements under the same fixed spatial outer folds: the original Sentinel-2 sequence followed by VV and VH, a modality-swapped sequence with VV and VH placed first, and one fixed random permutation of all 14 features. We then conducted an optical-only analysis in which VV and VH remained fixed in the final two positions, while the 12 Sentinel-2 bands were retained in the original order, reversed, or reordered using one fixed random permutation generated before model fitting and held constant across all folds.

In a separate matched analysis, the single-stream configuration was compared with an approximately parameter-matched modality-specific dual-branch alternative. The optical branch processed the 12 Sentinel-2 bands and produced a 128-dimensional latent representation, while an independent lightweight SAR branch processed VV and VH and produced a 16-dimensional representation. The two representations were concatenated before classification. The dual-branch model contained 236,757 trainable parameters, compared with 236,309 for IR-Spec1D, a difference of 0.19%. Across matched conditions, the cyclic 2/1/1/1 fold-role allocation, preprocessing, optimization settings, augmentation, early stopping, and fold-specific random seeds were held constant. All models were trained from scratch. These sensitivity analyses were restricted to source-domain classification and did not repeat probability calibration, TTA, scene-level inference, or transfer evaluation. The reserved calibration fold was not reassigned to training or validation.

#### 2.3.6 Training stochasticity, spatial partition, and paired-benchmark uncertainty

The standard deviations in the formal source-domain benchmark quantify fold-to-fold variation across the five spatially grouped held-out evaluation folds. Training stochasticity was evaluated separately by fitting the final IR-Spec1D configuration with five prespecified random seeds (42–46) while holding the spatial partition, cyclic 2/1/1/1 data roles, preprocessing, architecture, and hyperparameters fixed. Performance was first averaged across the five folds within each seed, after which variability was summarized across the five seed-level means.

Sensitivity to the particular group-to-fold allocation was assessed with five alternative group-disjoint five fold assignments of the same 80 fixed spatial groups. Each alternative partition retained the same cyclic 2/1/1/1 fold-role allocation. The IR-Spec1D architecture, preprocessing, hyperparameters, and training seed were fixed, and KNN was re-evaluated on the same alternative partitions. Matched uncertainty in the formal benchmark was estimated separately by a paired bootstrap of the 80 fixed spatial blocks. For each of 10,000 replicates (resampling seed 20260829), blocks were sampled with replacement, all pixels in a sampled block were retained, and the identical sampled blocks were used for IR-Spec1D and KNN. Percentile-based 95% confidence intervals were calculated for the paired differences in pooled cross-validated OA and macro-F1. The empirical bootstrap proportion with a positive difference is reported descriptively and is not interpreted as a conventional hypothesis-test *p*-value.

#### 2.3.7 Computational profiling

Computational cost was characterized in the revision profiling environment described above, using the same archived model definitions under a matched procedure. Trainable parameters and FP32 state-dictionary size were recorded directly, while multiply–accumulate operations (MACs) were measured with THOP and floating-point operations (FLOPs) were reported using the convention FLOPs = 2 × MACs. Central-processing-unit and GPU latency/throughput were measured after warm-up; GPU timing used CUDA synchronization, and peak allocated memory was recorded. The practical deployment benchmark directly executed the five fold-specific IR-Spec1D models with five perturbation passes per model (25 forward passes per valid pixel). Timing included fold-specific StandardScaler transformation, host-to-device transfer, Gaussian test-time perturbations, temperature scaling, Softmax, five-model probability averaging, argmax classification, and predictive-entropy calculation; disk input/output and map rendering were excluded. Operational time is additionally reported per one million valid pixels and, for reference, per square kilometer assuming a regular 10 m grid.

#### 2.3.8 Frozen-model temporal and closed-set geographic transfer evaluation

The frozen source-domain predictor comprised the five IR-Spec1D networks, their fold-specific StandardScalers, their fitted temperature-scaling parameters, the inference sequence in Equations 4–12b. These components were fixed before being applied to either target dataset. No target sample was used for model training, fine-tuning, configuration decisions, StandardScaler fitting, temperature-scaling parameter refitting, or model updating.

Same-site temporal transfer was evaluated with the April 2022 and April 2026 Dongzhai Harbor reference datasets. Each year-specific dataset contained 400 samples across the six source-domain categories. Locations were selected separately for each year and were not required to coincide. The samples were used only to evaluate the frozen source-domain predictor.

Closed-set geographic transfer was evaluated with the 288 Gaoqiao reference samples from the four categories prespecified as shared with the source taxonomy. Let *S* denote this prespecified category set. The six-category ensemble probabilities were restricted and renormalized as using Equation 16:

(16) π¯r,c(S)=π¯r,c∑j∈S π¯r,j, c∈S.

The four-category prediction map, predictive entropy, and sample-based metrics were all calculated from ${\bar{\pi }}_{r}^{\left(S\right)}$. The reference dataset comprised 32 field plots, nine adjacent samples per plot, and 72 samples per category. Gaoqiao samples did not contribute to source-domain training, fine-tuning, StandardScaler fitting, or temperature-scaling parameter estimation. The target region’s known shared category set was nevertheless used to define the inference label space, so the experiment is described as involving no target-domain training or adaptation, rather than as target-free.

To account for the clustered sampling structure of the Gaoqiao reference dataset, uncertainty in geographic-transfer performance was estimated using a class-stratified plot-level cluster bootstrap. Field plots, rather than individual reference samples, were used as the resampling units. Within each of the four shared reference categories, eight plots were sampled with replacement from the eight available plots, and all nine reference samples from each selected plot were retained together. OA, macro-F1, and class-specific precision, recall, and F1 were recalculated across 10,000 bootstrap replicates, and percentile-based 95% confidence intervals were obtained from the 2.5th and 97.5th percentiles. As a secondary diagnostic, the four frozen class probabilities were averaged across the nine samples in each plot before assigning the plot-level class by maximum mean probability.

To characterize geographic feature shift without confounding from label-space differences, the source-to-target comparison was restricted to the four taxa shared by Dongzhai Harbor and Gaoqiao. For each of the 14 input features, class-conditional source and target means were calculated separately for each shared taxon. Target-minus-source mean differences were standardized by the corresponding feature standard deviation calculated from the shared source-domain data and were interpreted as descriptive effect sizes rather than significance tests.

Nine samples from one field-observed *S. apetala* plot were examined separately and excluded from the four-category metrics. A single plot was insufficient to estimate unknown-species detection performance.

## 3 Results

Section 3 follows the primary evidence chain from source-domain classification to probability calibration and spatial predictive entropy. All source-domain models used the same input features, class definitions, spatial partitions, and evaluation metrics. Ablation, simulated feature omission, and frozen-model transfer were treated as supporting analyses.

### 3.1 Spatial grouping configuration for source-domain evaluation

Validation macro-F1 of the lightweight CNN proxy varied non-monotonically with the number of spatial groups (**Figure 3B**). Mean macro-F1 increased from *K* = 10 to *K* = 30, declined at *K* = 50, and reached its highest value at *K* = 80. Mean macro-F1 decreased at *K* = 120 and *K* = 200, with wider initialization variability at *K* = 200. The normalized-difference criterion also reached its maximum at *K* = 80 (**Figure 3C**).

The RF result at *K* = 80 was within 0.01 of the maximum RF macro-F1 across the tested candidates, providing a model-specific consistency check without determining the selected value. The selected *K* = 80 was treated as an empirical operating point rather than a statistical optimum. It defined the grouping granularity used in the subsequent fivefold spatially grouped evaluation and was not itself a final performance estimate. The resulting *K* = 80 grouping retained all six categories in every held-out evaluation fold and was fixed for all subsequent analyses.

The ROI-level integrity audit confirmed that all 373 reference ROI units were confined to a single outer fold (373/373; 0 split). The pixel/group audit likewise confirmed that all 80 coordinate-defined spatial groups were preserved during outer-fold assignment (0/80 split), with no duplicated reference-pixel coordinates across folds. Under the primary protocol, the minimum training-to-evaluation distance was 10 m in every fold, and the median nearest-training distances were 30.0, 31.6, 28.3, 33.8, and 30.0 m for folds 1–5. Among the 3,327 cross-validated observations, 92 were misclassified (2.77%); the pooled binary error indicator yielded Moran’s *I* = 0.4968 under the primary eight-nearest-neighbor definition (9,999 permutations; *p<* 0.001), with alternative spatial-weight checks reported in **Supplementary Table S8**.

In the buffered sensitivity analysis, the actual minimum training-to-evaluation separation increased to 22.36, 50.99, and 100.50 m for the 20-, 50-, and 100-m exclusion radii, respectively. These exclusions removed an average of 10.7%, 30.9%, and 51.4% of training observations, while pooled OA/macro-F1 values remained 0.9594/0.9096, 0.9441/0.8949, and 0.9318/0.8929. In the matched 50-m buffered comparison, IR-Spec1D retained the highest point estimates of pooled OA and macro-F1 (0.9441 and 0.8949), compared with KNN (0.9399/0.8751), RF (0.9161/0.8891), and 1D-ConvNeXt (0.9122/0.8015). Full fold-wise audit and buffer results are reported in **Supplementary Tables S7**–**S10**.

### 3.2 Source-domain classification and probability calibration

Under fivefold spatially grouped cross-validation, IR-Spec1D achieved a mean OA of 0.9720 and a mean macro-F1 of 0.9406 (**Figure 5A**). Fold-level OA ranged from 0.9513 to 0.9970, and macro-F1 ranged from 0.8925 to 0.9972.

**Figure 5:**
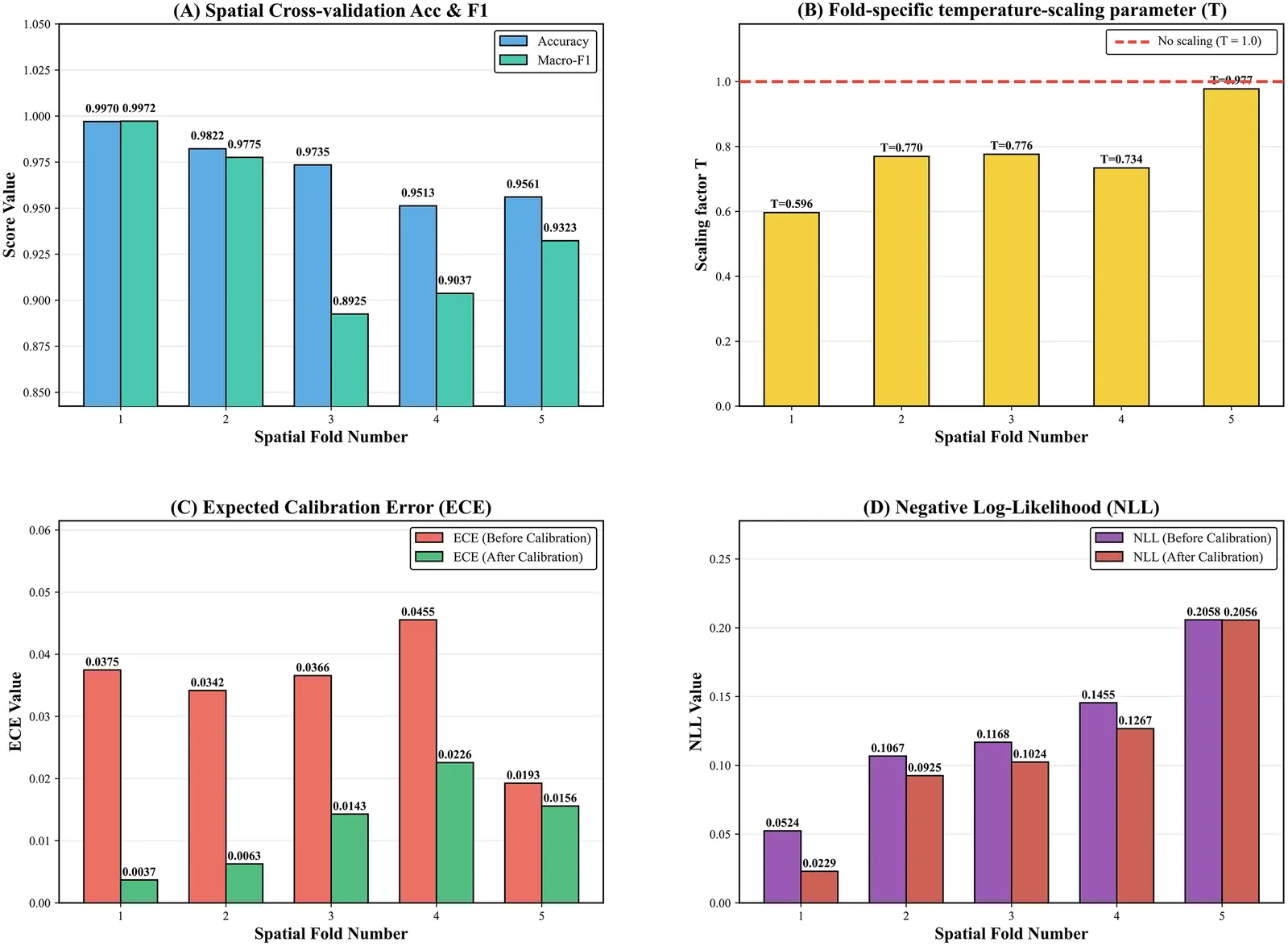
Source-domain performance and probability calibration. **(A)** OA and macro-F1. **(B)** Fitted temperature-scaling parameter. **(C)** ECE before and after temperature scaling. **(D)** NLL before and after temperature scaling. OA, macro-F1, ECE, and NLL were evaluated on the held-out outer folds $D_{f}^{\text{eval}}$; the parameter in **(B)** was fitted on the separate calibration subset $D_{f}^{\text{cal}}$.

Producer’s accuracy exceeded 0.98 for *R. stylosa*, *C. tagal*, and *K. obovata*, with F1 scores from 0.9784 to 0.9929. The *Bruguiera* complex and *A. corniculatum* had F1 scores of 0.8892 and 0.8986, respectively; these category-level results are also shown in the IR-Spec1D column of **Figure 6**.

**Figure 6:**
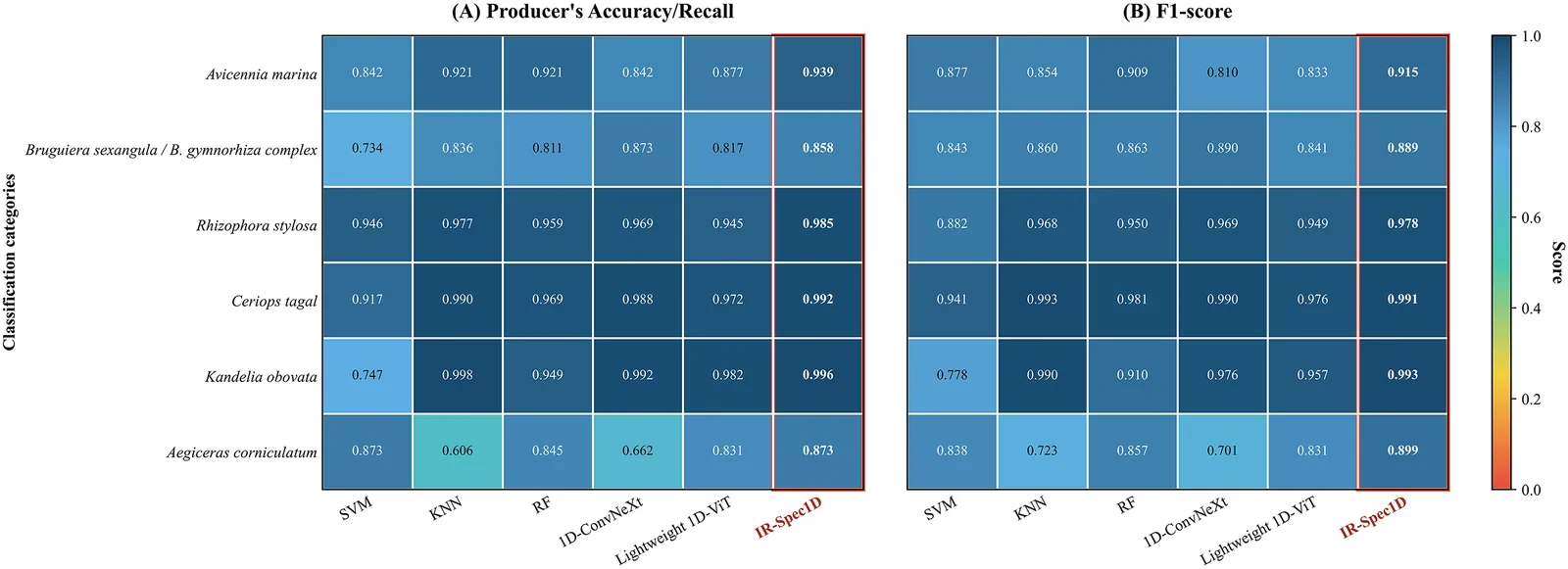
Category-level model performance. **(A)** Producer’s accuracy (recall). **(B)** F1 score. Red frames identify IR-Spec1D.

The fitted temperature-scaling parameters were below 1 in all five folds, with a mean of 0.7707 (**Figure 5B**). Under the stated regularized fitting procedure, the transformation sharpened rather than softened the probability distributions without changing the predicted labels. Mean ECE decreased from 0.0346 to 0.0125 (**Figure 5C**), while mean NLL decreased from 0.1254 to 0.1100 (**Figure 5D**) These reductions describe probability calibration on the held-out evaluation folds after fitting the temperature-scaling parameter on the separate calibration subsets. The temperature-scaled probabilities were subsequently used to compute predictive entropy.

### 3.3 Class-balanced performance relative to matched benchmarks

IR-Spec1D was compared with five benchmark models under matched spatial folds.

IR-Spec1D achieved the highest mean value for both metrics (**Table 2**). KNN had the next-highest mean OA at 0.9601, an absolute difference of 0.0119, or 1.19 percentage points. RF had the next-highest mean macro-F1 at 0.8730, an absolute difference of 0.0676, or 6.76 percentage points.

Metric rankings differed. KNN ranked second in OA and fourth in macro-F1, whereas RF ranked fourth in OA and second in macro-F1.

The category-level matrices further resolved these differences (**Figure 6**). IR-Spec1D had the highest F1 score in four of the six categories; it did not have the highest F1 score for *C. tagal* or the *Bruguiera* complex.

Producer’s accuracy for *A. corniculatum* was 0.6056 with KNN and 0.6620 with 1D-ConvNeXt. IR-Spec1D category-level F1 scores ranged from 0.8892 to 0.9929. It did not rank first for every category–metric combination.

Relative to the matched benchmarks, the advantage of IR-Spec1D was more pronounced for macro-F1 than for OA. The difference in OA from KNN was 1.19 percentage points, whereas the difference in macro-F1 from RF was 6.76 percentage points. This pattern indicates that the principal aggregate gain lay in more balanced performance across categories rather than uniform dominance in every category–metric combination. The different rankings of KNN and RF further show why OA and macro-F1 must be interpreted together under the imbalanced source-domain category distribution.

The formal values reported as mean ± standard deviation in **Table 2** represent fold-to-fold variation and do not represent uncertainty from repeated-run training stochasticity. Across five prespecified training seeds under the fixed spatial partition, the fivefold mean OA and macro-F1 at the seed-level were 0.9727 ± 0.0010 and 0.9285 ± 0.0196, respectively, where the standard deviations represent seed-to-seed variability. Across five alternative group-disjoint fivefold assignments of the same 80 fixed spatial groups, IR-Spec1D achieved mean OA = 0.9723 ± 0.0019 and macro-F1 = 0.9432 ± 0.0044. The corresponding IR-Spec1D minus KNN partition-level differences were + 0.0122 ± 0.0016 in OA and + 0.0493 ± 0.0062 in macro-F1, and both aggregate differences were positive in all five alternative partitions. A separate paired spatial-block bootstrap using the formal benchmark yielded a pooled cross-validated OA difference of + 0.0117 (95% CI, 0.0031 to 0.0244) and a macro-F1 difference of + 0.0462 (95% CI, 0.0032 to 0.1186). **Supplementary Tables S14**-**S16** distinguish the complete seed, partition, and paired-bootstrap summaries.

The input-order and fusion controls directly tested the assumption underlying one-dimensional processing of the heterogeneous vector. Relative to the matched original configuration, moving the Sentinel-1 VV/VH block to the front changed mean fold OA and macro-F1 by + 0.0076 and + 0.0030, while one fixed random permutation of all 14 features changed them by + 0.0022 and − 0.0004. In the optical-only analysis, reversing the order of the 12 Sentinel-2 bands produced pooled differences of − 0.0018 in OA and − 0.0059 in macro-F1, with 95% spatial-block bootstrap CIs of − 0.0075 to + 0.0035 and − 0.0294 to + 0.0075. The fixed random optical-band permutation produced pooled differences of − 0.0003 and + 0.0003, with corresponding CIs of − 0.0090 to + 0.0074 and − 0.0339 to + 0.0249. The approximately parameter-matched modality-specific dual-branch alternative likewise showed no consistent performance advantage: its pooled differences relative to the matched input-level configuration were − 0.0039 in OA and − 0.0011 in macro-F1, with CIs of − 0.0098 to + 0.0012 and − 0.0140 to + 0.0102. Together with the Sentinel-1 ablation reported below, these results support limited sensitivity to the tested arrangements without implying order invariance, physical continuity of the inputs, or physical optimality of the single-stream design (**Supplementary Tables S4**, **S5**).

### 3.4 Predictive entropy and field/UAV-informed spatial interpretation

For each classifier, the single full-scene map in **Figure 7** was generated from the corresponding five fold-specific models retrained under the matched protocol, rather than from one selected fold. The maps were used to examine how the sample-based results were represented spatially and were interpreted qualitatively. SVM, KNN, and RF produced visually fragmented labels in several areas, and the KNN map remained fragmented despite its second-highest mean OA. The 1D-ConvNeXt and Lightweight 1D-ViT outputs contained broader contiguous regions but differed in transition zones and small marginal patches.

**Figure 7:**
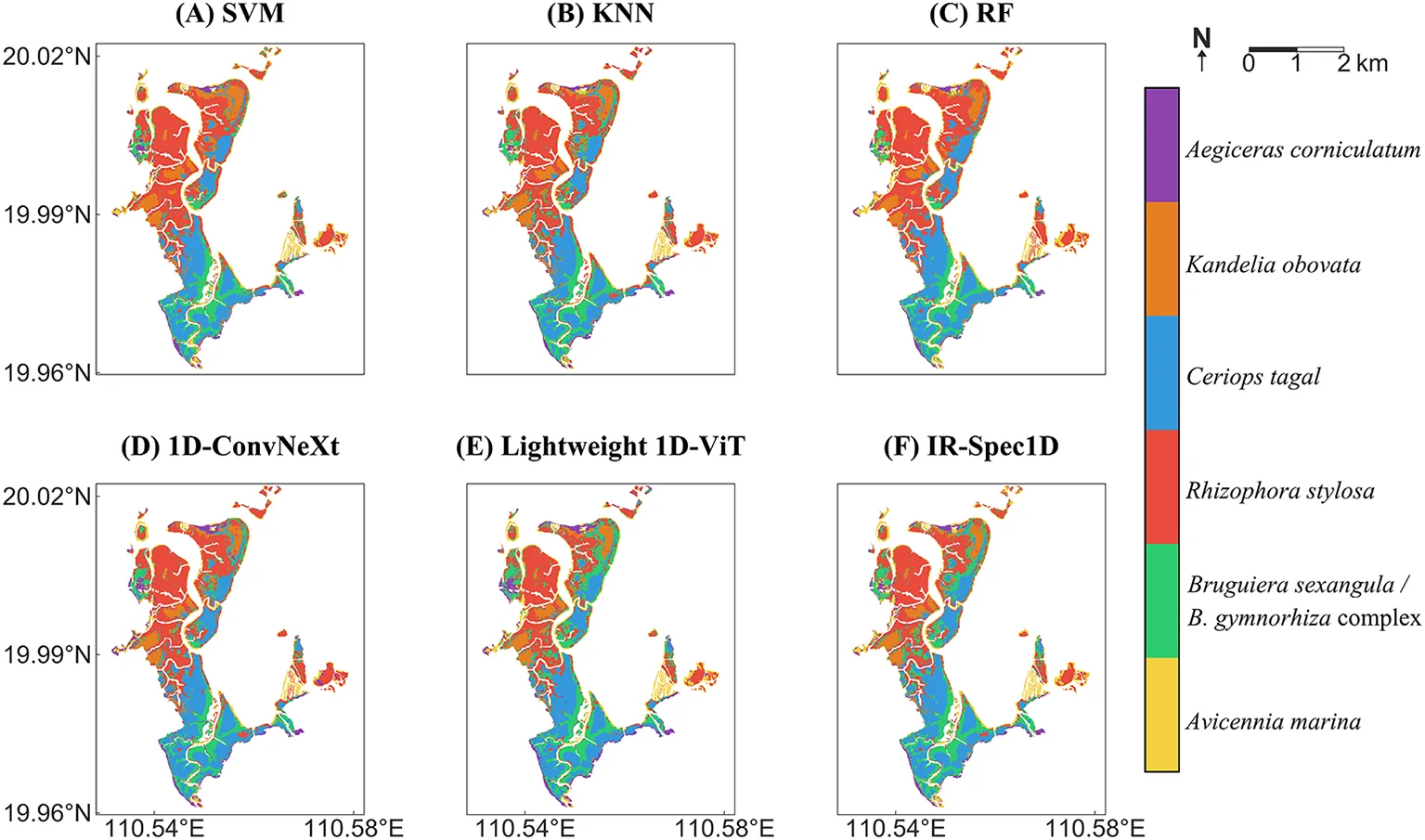
Spatial comparison of model predictions. **(A–F)** SVM, KNN, RF, 1D-ConvNeXt, Lightweight 1D-ViT, and IR-Spec1D, respectively. Each full-scene map was produced from the corresponding five fold-specific models, not from a selected single fold.

IR-Spec1D appeared more spatially contiguous than several comparison outputs in the inspected maps. Broad interior areas were assigned to *R. stylosa* and *C. tagal*, while narrower marginal areas were assigned to *A. corniculatum* and *A. marina*. Given that all six classifiers used pixel-level feature vectors without explicit neighborhood inputs, these spatial patterns reflect differences in their learned feature-based decisions rather than direct spatial-context modeling.

IR-Spec1D produced both maximum-probability category labels and predictive entropy from the temperature-scaled ensemble probabilities (**Figure 8**). The entropy map preserves information about class-probability dispersion that is absent from the categorical label map, but visual patterns alone were not used to infer errors or ecological boundaries.

**Figure 8:**
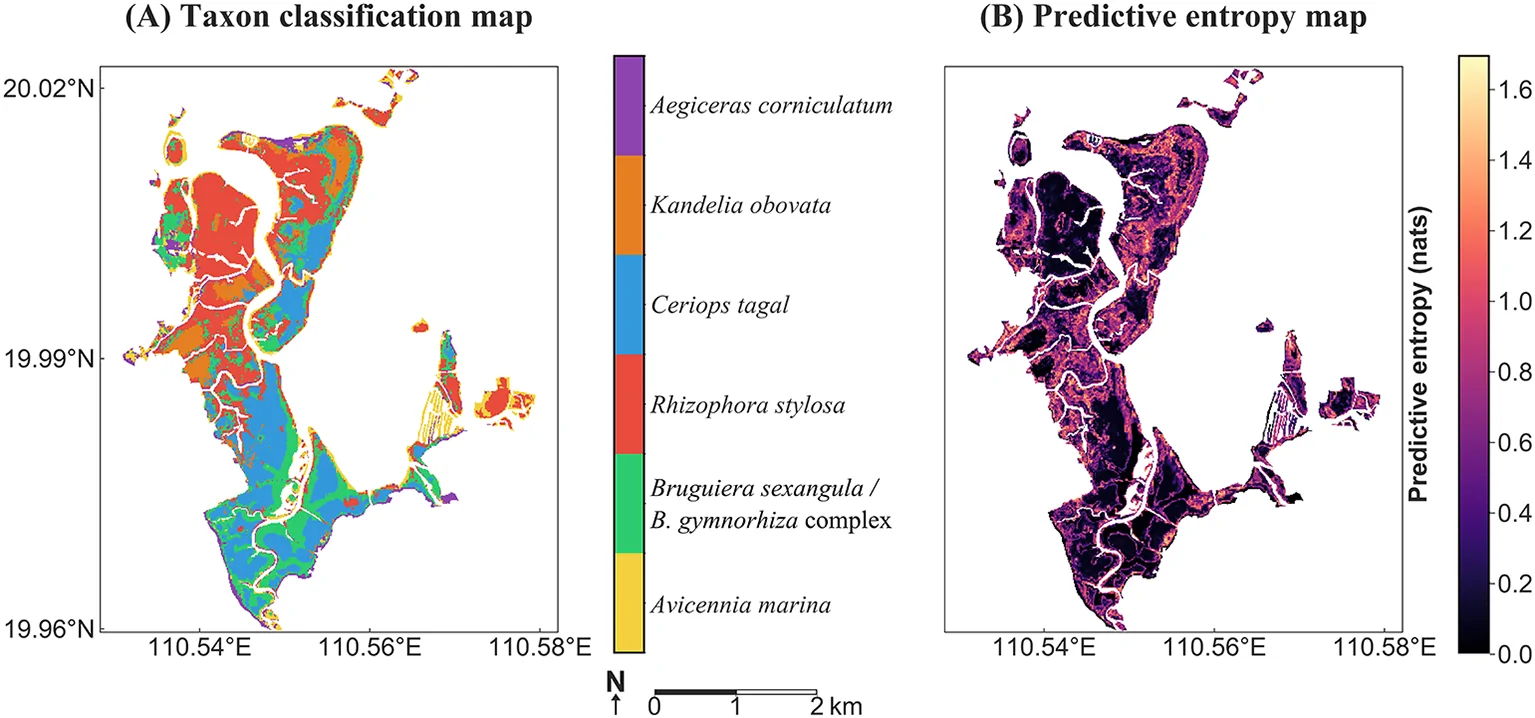
Source-domain classification and predictive entropy. **(A)** Taxon-classification map. **(B)** Predictive-entropy map derived from temperature-scaled ensemble probabilities. Entropy is expressed in nats.

Predictive entropy derived from the temperature-scaled probabilities was strongly associated with cross-validated classification error. The 92 misclassified observations had a mean entropy of 0.5861 nats, compared with 0.0964 nats for the 3,235 correct observations. The difference between misclassified and correctly classified observations was 0.4897 nats (95% CI, 0.3066–0.7457). Predictive entropy ranked cross-validated errors with an AUROC of 0.8808 (95% CI, 0.7657–0.9499) and an AUPRC of 0.3884 (95% CI, 0.1582–0.5839), compared with an error prevalence of 0.0277. The highest entropy quartile captured 88.04% of all errors and exhibited a 22.08-fold higher error rate than the lower three quartiles (95% CI, 7.52–92.53).

Boundary distance itself showed no clear monotonic association with predictive entropy (Spearman *ρ* = 0.0064; 95% CI, − 0.1624 to 0.1706), but boundary proximity identified an error-prone spatial context: the cross-validated error rate was 11.81% within 20 m of the mapped mangrove boundary and 1.58% beyond 50 m, corresponding to a 7.50-fold error-rate ratio (95% CI, 1.60–31.42). The Sentinel-2 heterogeneity analysis likewise did not show a simple monotonic rank relationship across the full distribution (Spearman *ρ* = 0.0732; 95% CI, − 0.0690 to 0.2097), while the prespecified highest-heterogeneity quartile showed a 0.0982-nat increase in mean predictive entropy relative to Q1 (95% CI, 0.0418–0.1595), and the error rate increased from 0.48% in Q1 to 8.89% in Q4. The complete validation summary is provided in **Supplementary Table S6**.

**Figure 4** relates all three case regions to reference interpretations established independently of the models. In Region 1, field and orthophoto evidence documented an *R. stylosa*-dominated stand with a mixed canopy. Region 2 was a mixed planting strip in which the outlined focal plot was dominated by *A. corniculatum*, with *R. stylosa* and *A. marina* also present. Region 3 contained an *R. stylosa* core with possible *C. tagal* occurrence toward the margins. The coregistered focal boxes permit qualitative comparison of local model behavior with this independent evidence; they do not constitute additional numerical validation samples.

These cases show that similar aggregate metrics can coexist with different local label configurations. The paired classification and predictive-entropy maps provide complementary spatial information: the former records the most probable modeled taxon, whereas the latter identifies locations where probability mass is more dispersed among competing taxa. In a prespecified shared label space, comparatively high-entropy locations can therefore be prioritized for closer inspection, while the quantitative cross-validated analysis above—rather than visual appearance alone—supports this interpretation for error prioritization.

### 3.5 Ablation and sensitivity to simulated feature omission

Two analyses examined the model configuration. Leave-one-component-out ablations quantified changes in OA and macro-F1 under the matched *K* = 80 spatially grouped cross-validation protocol (**Figure 9**). A separate experiment compared the complete model with the variant without Spectral-Domain Dropout under simulated random feature omission (**Figure 10**).

**Figure 9:**
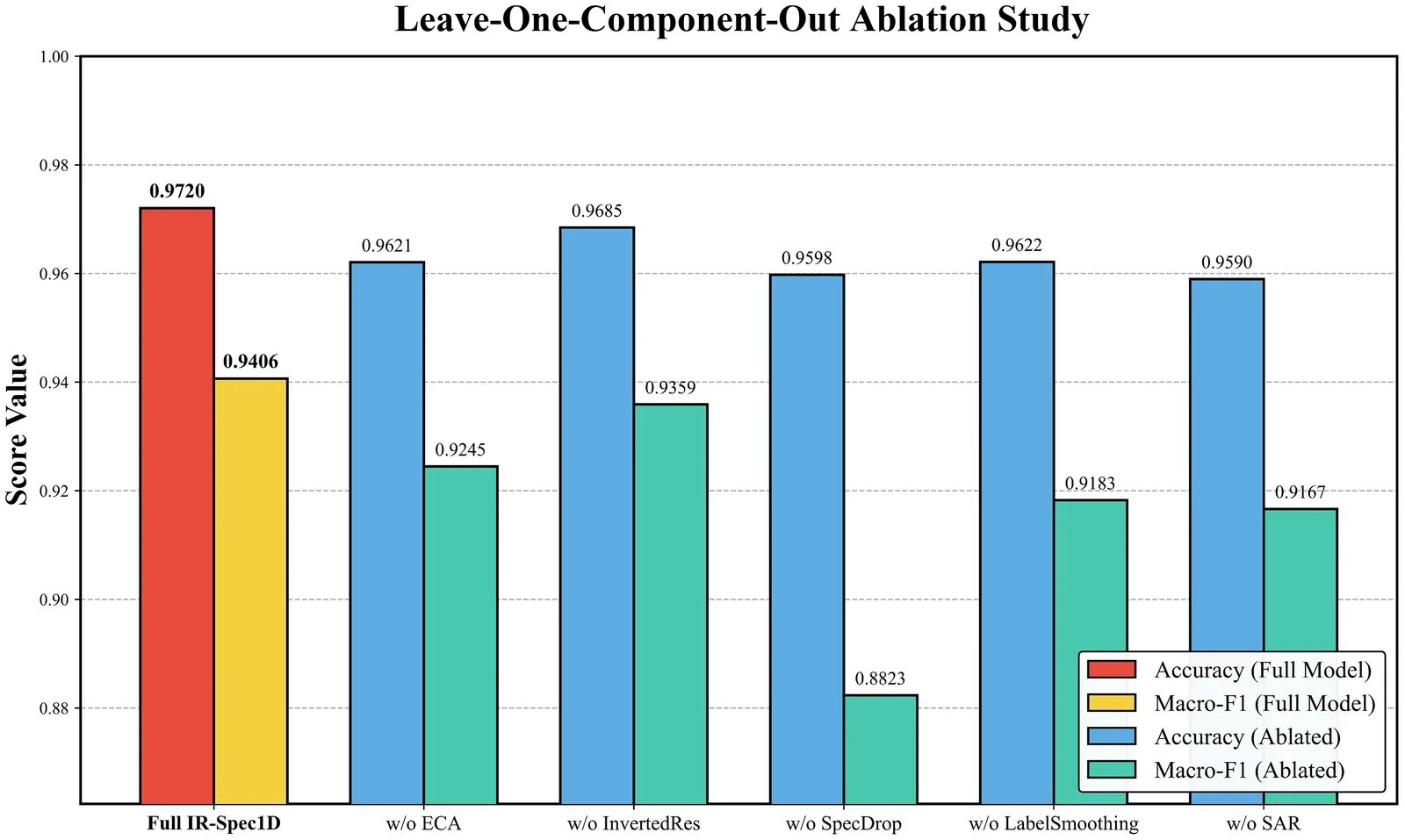
Leave-one-component-out ablation of IR-Spec1D. Mean OA and macro-F1 for the complete model and component-removal variants under matched spatially grouped evaluation at *K* = 80.

**Figure 10:**
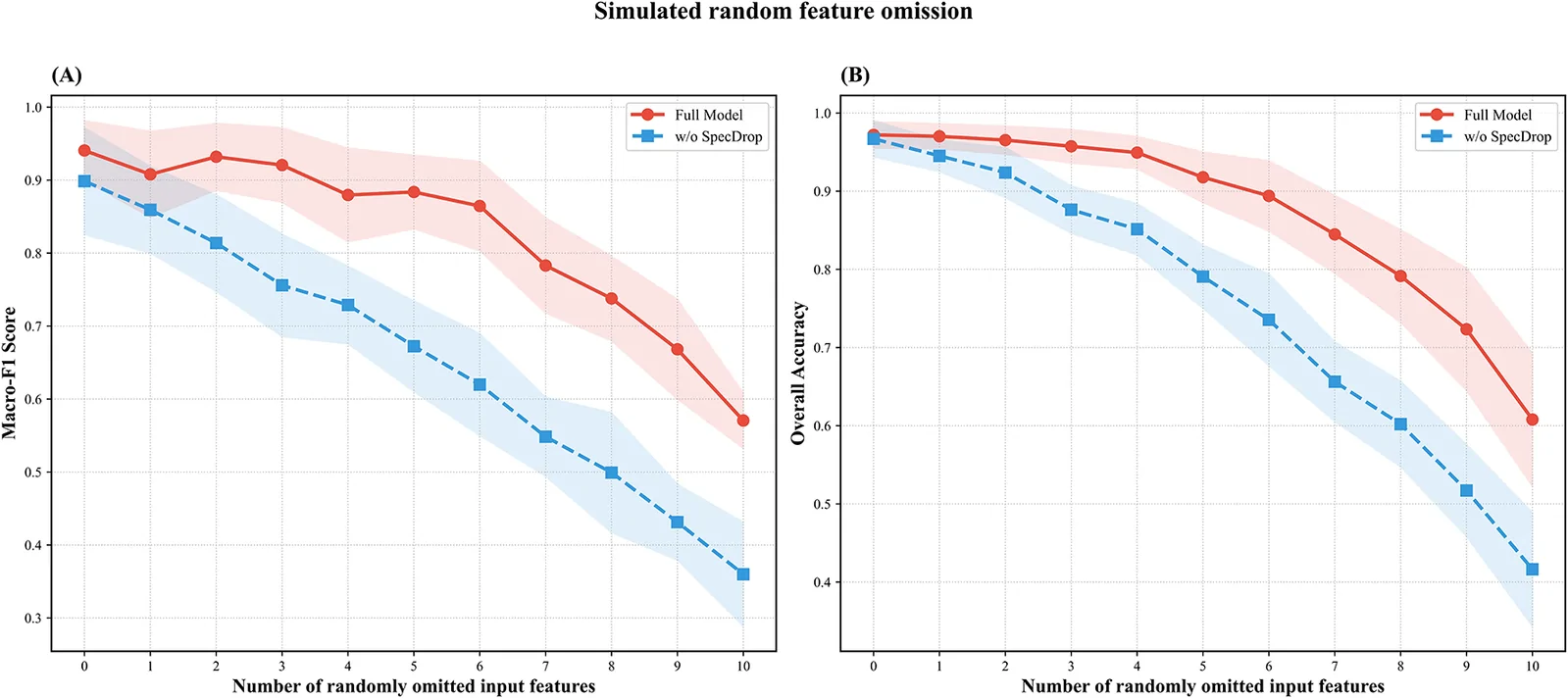
Sensitivity to simulated feature omission. Curves compare the complete model with the variant without Spectral-Domain Dropout. **(A)** Macro-F1. **(B)** OA. Curves show means across five fold-level values after within-fold averaging over five sampled omission subsets; shaded bands denote ± 1 standard deviation across these fold-level values.

The complete model achieved an OA of 0.9720 and a macro-F1 of 0.9406 (**Figure 9**). Removing the Sentinel-1 inputs reduced OA to 0.9590 and macro-F1 to 0.9167. Removing Efficient Channel Attention yielded an OA of 0.9621 and a macro-F1 of 0.9245, while removing the Inverted Residual blocks yielded an OA of 0.9685 and a macro-F1 of 0.9359. Without Label Smoothing, OA was 0.9622, and macro-F1 was 0.9183. These comparisons quantify the performance change associated with each removal under the tested configuration.

Removing Spectral-Domain Dropout yielded an OA of 0.9598 and a macro-F1 of 0.8823. This was the largest macro-F1 decrease among the ablations, an absolute reduction of 0.0583 from the complete model.

OA and macro-F1 decreased as more features were replaced by their training-subset means (**Figure 10**). The complete model remained above the variant without Spectral-Domain Dropout across the evaluated omission levels. With up to five omitted features, the complete model retained an OA above 0.90 and a macro-F1 above 0.85.

### 3.6 Mapping-footprint sensitivity and computational profile

Across the four domain–year maps, the dominant mapped taxon was unchanged after both the 10- and 20-m inward boundary sensitivity tests. After 20 m erosion, the largest absolute change in any category proportion was 4.85 percentage points in Dongzhai Harbor 2022, 6.60 percentage points in Dongzhai Harbor 2024, 3.31 percentage points in Dongzhai Harbor 2026, and 3.37 percentage points in Gaoqiao 2024. The sensitivity was therefore concentrated in the proportional contribution of particular edge-associated categories rather than in a reversal of the dominant interior taxonomic composition. Counts and category-composition summaries are reported in **Supplementary Table S2**.

In the revision profiling environment, IR-Spec1D contained 236,309 trainable parameters, corresponding to a 0.9530-MB FP32 state dictionary, and required 1.219648 MMACs (2.439296 MFLOPs under the 2 × MAC convention) per 14-feature sample. On the RTX 4090, batch-1 latency was 1.3557 ms and batch-4096 throughput was 486,956 samples s^−1^, with 93.98 MB peak allocated GPU memory. Direct profiling of the complete five-model × five-perturbation workflow yielded 15,001.72 valid pixels s^−1^, equivalent to 66.659 s per million valid pixels or 0.6666 s km^−2^ under a regular 10-m grid conversion, with 93.82 MB peak allocated GPU memory. The corresponding direct CPU throughput was 1,200.57 valid pixels s^−1^. Matched model-complexity metrics and CPU/GPU values are reported in **Supplementary Tables S12**, **S13**.

### 3.7 Auxiliary frozen-model transfer evaluation

As an auxiliary evaluation, the trained networks, fold-specific scalers, and temperature-scaling parameters were frozen before being applied to the two temporal datasets and Gaoqiao. These experiments assessed performance retention under the specified target settings.

#### 3.7.1 Same-site temporal transfer

The frozen ensemble correctly classified 378 of 400 reference samples in each target year, yielding an OA of 0.9450 in both cases (**Table 3**). Macro-F1 was 0.9411 in 2022 and 0.9402 in 2026. ECE was 0.0359 in 2022 and 0.0511 in 2026.

**Table 3:** Temporal and closed-set geographic performance of the frozen IR-Spec1D ensemble.

Target region	Year	Evaluation setting	Classes	*N*	OA	Macro-F1
Dongzhai Harbor	2022	Temporal transfer	6	400	0.9450	0.9411
Dongzhai Harbor	2026	Temporal transfer	6	400	0.9450	0.9402
Gaoqiao	2024	Closed-set geographic transfer	4	288	0.7118	0.7131

*N* denotes the number of reference samples used for numerical evaluation.

The mapped category patterns were similar at broad scales, with localized differences near narrow margins and fragmented patches (**Figure 11**). In 2022, the *Bruguiera* complex had the highest F1 score of 0.983. *K. obovata* had the lowest F1 score of 0.896 and a recall of 0.833.

**Figure 11:**
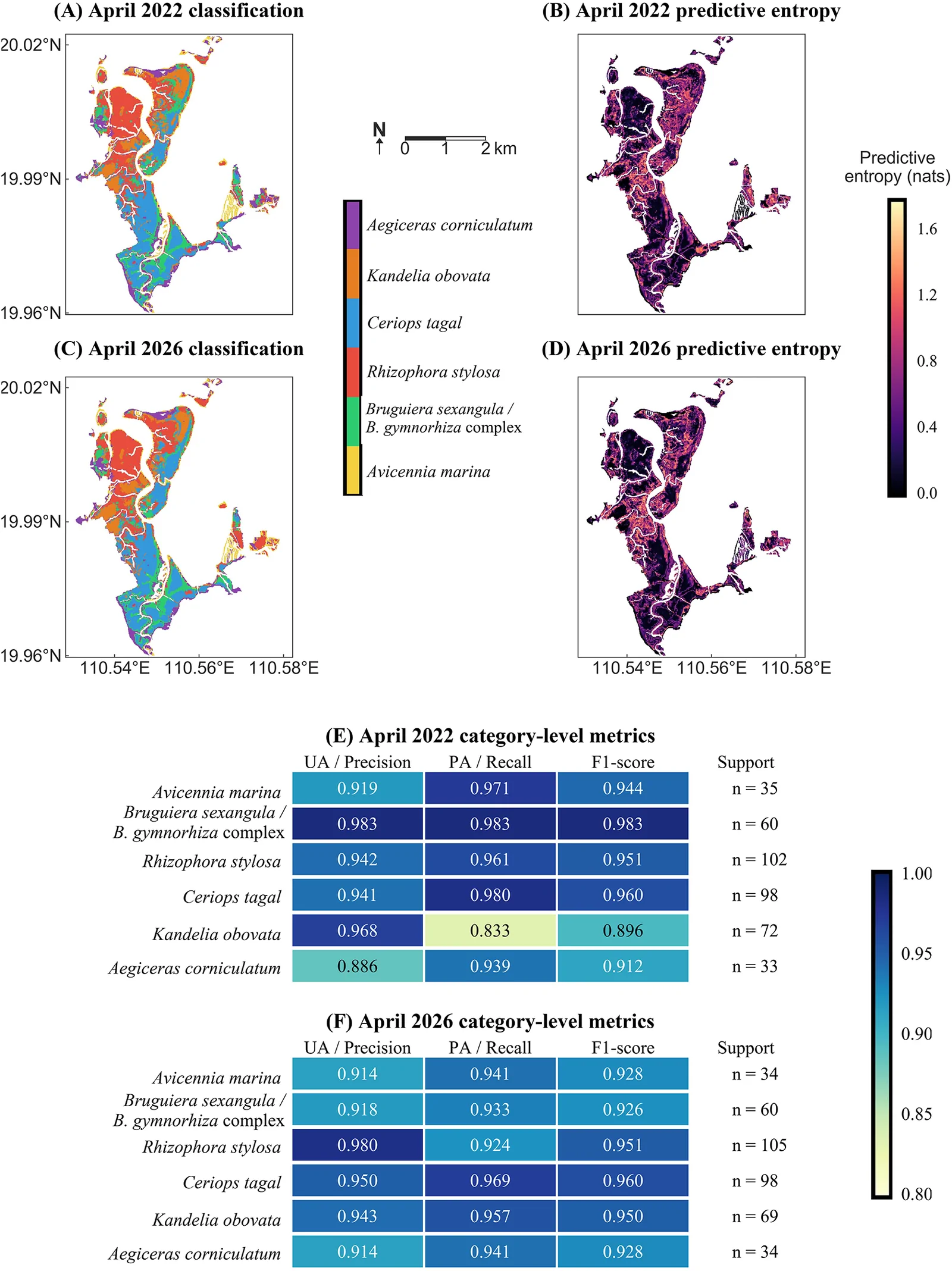
Frozen temporal transfer in Dongzhai Harbor. **(A, B)** Taxon classification and predictive entropy maps for April 2022. **(C, D)** Corresponding maps for April 2026. **(E, F)** Category-level user’s accuracy (precision), producer’s accuracy (recall), F1 score, and support. Support denotes the number of year-specific reference samples.

In 2026, category-level F1 scores ranged from 0.926 to 0.960.

#### 3.7.2 Closed-set geographic transfer to Gaoqiao

The Gaoqiao numerical evaluation used 288 reference samples from the four categories shared with the source taxonomy. Field-observed *S. apetala* references were retained separately and were not included in the four-category closed-set accuracy metrics. The frozen evaluation yielded 205 correct predictions, corresponding to an OA of 0.7118 with a plot-cluster bootstrap 95% CI of 0.5799 to 0.8368 and a macro-F1 of 0.7131 with a 95% CI of 0.5754 to 0.8354. The four-category prediction map, predictive entropy map, and category-level metrics are shown in **Figure 12**. Class-specific F1 estimates (95% plot-cluster CIs) were 0.6533 (0.5610–0.7482) for *A. marina*, 0.6970 (0.5846–0.8000) for the *Bruguiera* complex, 0.6133 (0.5098–0.7051) for *R. stylosa*, and 0.8889 (0.8085–0.9524) for *A. corniculatum*. Full class-specific precision, recall, and F1 intervals are reported in **Supplementary Table S17**. As a secondary plot-level diagnostic, mean-probability aggregation across the nine samples within each field plot correctly classified 25 of 32 plots (OA = 0.7813, macro-F1 = 0.7793, Cohen’s *κ* = 0.7083; **Supplementary Table S18**).

**Figure 12:**
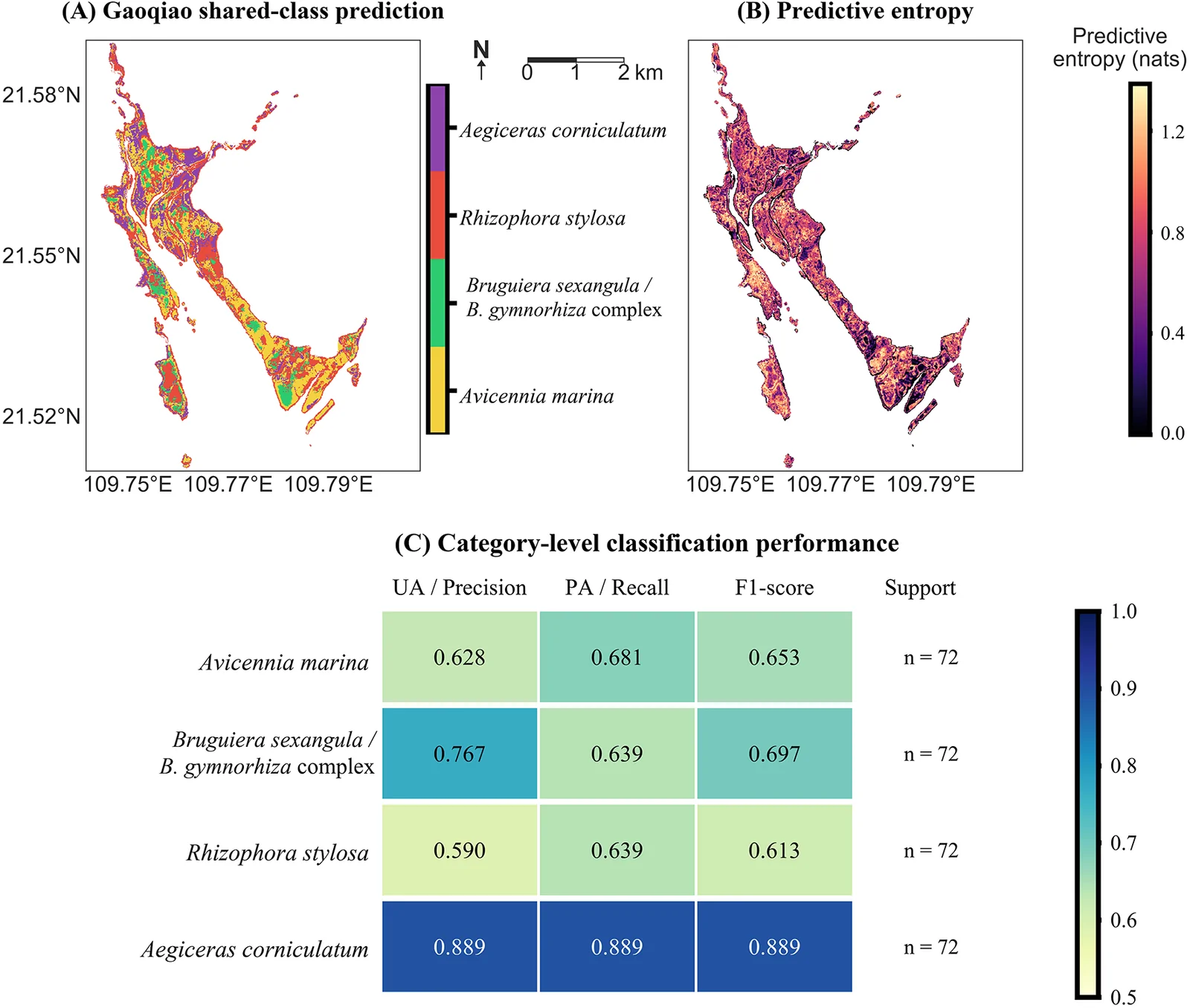
Closed-set geographic transfer to Gaoqiao. Frozen source-domain ensemble probabilities were restricted and renormalized to the four categories prespecified as shared with Gaoqiao. No Gaoqiao reference sample was used for training, fine-tuning, StandardScaler fitting, temperature-scaling parameter refitting, or model updating; the known shared category set nevertheless defined the closed-set inference label space. **(A)** Four-category taxon classification map. **(B)** Predictive entropy map. **(C)** Category-level user’s accuracy (precision), producer’s accuracy (recall), F1 score, and support for the 288 shared-category reference samples. Field-observed *S. apetala* references were retained separately and excluded from the four-category closed-set metrics.

The Gaoqiao sample-level point estimates are accompanied by class-stratified plot-cluster bootstrap 95% CIs of 0.5799–0.8368 for OA and 0.5754–0.8354 for macro-F1; the field plot, rather than adjacent samples, was the resampling unit.

A class-matched feature comparison showed a clear geographic shift between Dongzhai Harbor (*n* = 1,749 shared-class observations) and Gaoqiao (*n* = 288). Across all four shared taxa, Gaoqiao values were higher for Sentinel-2 B1, B2, B3, B4, B5, B6, B7, B8, B8A, and B9. The strongest mean standardized shifts occurred in B2, B1, B3, B4, and B5. The remaining four features, B11, B12, VV, and VH, showed mixed shift directions across taxa. **Supplementary Table S21** reports the mean signed and mean absolute standardized shifts for all 14 input features. The lower Gaoqiao accuracy was therefore observed alongside a measurable class-matched cross-region radiometric and spectral feature shift under a fully frozen setting. This analysis does not isolate a single causal mechanism for the decrease.

Relative to the mean of the two same-site temporal evaluations, Gaoqiao OA was 23.32 percentage points lower, and macro-F1 was 22.76 percentage points lower. These numerical differences are descriptive because the settings differed in label space, sampling design, and category composition.

## 4 Discussion

### 4.1 Class-balanced classification under spatially grouped evaluation

IR-Spec1D achieved the highest mean OA and macro-F1 among six models under the matched five-fold spatially grouped protocol. Its larger advantage in macro-F1 indicates improved aggregate class-balanced discrimination across the unevenly represented categories, although it did not lead in every category–metric comparison. This result complements mangrove studies using fine-resolution observations, synthesized Sentinel-2 imagery, and multisensor or ensemble learning (Lassalle et al., 2023; Fu et al., 2023, 2024; Zhao et al., 2024). The contribution lies in the task-specific configuration and evaluation of established operations for heterogeneous optical and SAR inputs.

All 373 reference ROIs and 80 spatial groups remained intact within folds, with no duplicated reference pixel coordinates across folds. Spatial clustering nevertheless remained in the cross-validated source-domain errors. Preserving the original reference units prevents cross-fold overlap, while the distance audit and buffers assess sensitivity to nearby training observations. Exclusion buffers jointly increased training–evaluation separation and reduced training data, so their effects cannot be attributed to separation alone. At the 100-m buffer, pooled OA and macro-F1 remained 0.9318 and 0.8929. IR-Spec1D also retained the highest point estimates in the matched 50 m comparison. These results indicate that short-range proximity alone is unlikely to explain the principal source-domain pattern, although this interpretation remains conditional on the sampled landscape.

The aggregate comparison persisted across five training seeds and five alternative group-to-fold assignments. IR-Spec1D–KNN differences were positive in every alternative assignment, and paired 80-block bootstrap intervals excluded zero for both pooled OA and macro-F1 differences. Seed variation characterizes optimization under a fixed partition; alternative fold assignments test group allocation; and paired block resampling preserves the matched model comparison. Together, these analyses support stability beyond a single initialization or fold assignment, while uncertainty remains for sparsely represented categories and unobserved environments. These comparisons remain conditional on the data-informed *K* = 80 grouping; the sensitivity analyses do not constitute nested selection of *K*.

Removing Sentinel-1 reduced OA and macro-F1, supporting the contribution of radar information within the tested configuration. Global and optical-only rearrangements produced small changes, and the optical-only confidence intervals included zero. The approximately parameter-matched dual-branch likewise showed no consistent advantage over input-level processing. These controls support limited sensitivity to the tested feature arrangements and characterize learned transformations over an ordered heterogeneous vector, without establishing a continuous wavelength interpretation or an optimal fusion design.

Field and UAV case comparisons show how similar aggregate metrics can coexist with different local category arrangements. These cases provide ecological context rather than additional accuracy samples (McRoberts, 2011). The dominant mapped taxon remained unchanged under 10 and 20 m inward mask contractions in all four domain–year maps. Changes in individual category proportions nevertheless show that footprint delineation affects area estimates, particularly along narrow fringes and mixed boundary pixels.

In the revision profiling environment, the complete 25-pass workflow required approximately 66.7 s per million valid pixels and less than 100 MB of peak allocated GPU memory. The profile includes scaling, perturbation, probability averaging, classification, and entropy computation, but excludes disk input/output and map rendering. These measurements support practical execution under the reported hardware and processing conditions. Model size and the matched computational profiles provide the basis for characterizing IR-Spec1D as compact.

### 4.2 Source-domain calibration and predictive entropy

Temperature scaling acts after network training, whereas Spectral-Domain Dropout and Gaussian perturbations regularize training inputs. Within each outer iteration, two folds supported model fitting and one fold supported validation-loss monitoring, early stopping, and checkpoint selection under fixed settings. A separate calibration fold supported the fitting of the temperature-scaling parameter, while the held-out evaluation fold supplied the reported probability diagnostics. Mean 15-bin ECE decreased from 0.0346 to 0.0125, and mean NLL decreased from 0.1254 to 0.1100 without changing categorical decisions. These held-out source-domain diagnostics describe the probability adjustment separately from classification accuracy (Guo et al., 2017; Balanya et al., 2024). The fitted scaling parameters were then frozen for scene-level and transfer inference.

Predictive entropy ranked cross-validated source-domain errors with an AUROC of 0.8808; its highest quartile contained 88.04% of those errors. This association supports its use as an inspection cue within the evaluated source-domain setting. Neither boundary distance nor local Sentinel-2 heterogeneity showed a simple monotonic relationship with entropy. Errors were more frequent near the boundary, however, and the highest-heterogeneity quartile was more difficult than the lowest. These findings provide contextual associations without identifying deterministic causes or establishing a universal error threshold. Predictive entropy remains a measure of class-probability dispersion and was not evaluated as an open-set rejection criterion.

### 4.3 Frozen temporal and closed-set geographic transfer

The frozen ensemble achieved an OA of 0.9450 in both the April 2022 and April 2026 Dongzhai Harbor evaluations. Each year used different reference locations and one acquisition per sensor, so this evidence concerns performance retention for those observations.

Gaoqiao yielded lower OA and macro-F1, with values of 0.7118 and 0.7131. Its 288 reference observations originated from 32 plots, which served as the resampling units for uncertainty estimation. Plot-cluster intervals retain the within-plot grouping, while the secondary 32-plot probability-aggregation diagnostic summarizes predictions at the field sampling unit. Class-matched feature comparisons documented a shifted target distribution, with the largest standardized differences in Sentinel-2 optical features. This shift accompanies the accuracy decrease, but geography, acquisition conditions, reference design, and label composition also differed.

The four Gaoqiao categories were prespecified before inference, and source probabilities were restricted and renormalized to that shared label space. Networks, scalers, and temperature-scaling parameters remained fixed, and no target reference observation was used for fitting or adaptation. Earlier locally trained Gaoqiao models used different data and validation protocols and provide context rather than a matched benchmark (Gao et al., 2022).

### 4.4 Scope and priorities for further validation

Broader portability requires additional independently sampled regions and acquisitions beyond the present source region and geographic target. The mapped label spaces contain six source categories and four prespecified Gaoqiao categories; unknown-taxonomy deployment and open-set recognition were not evaluated. Adding *S. apetala* requires source references meeting the same quality-control standard, while the *B. sexangula*/*B. gymnorhiza* complex retains the supported taxonomic resolution. Feature masking and Gaussian perturbations operate in standardized feature space, so the omission results concern the tested perturbations rather than physical cloud effects or sensor-degradation processes.

## 5 Conclusion

IR-Spec1D combines a compact optical–SAR classifier with spatial validation, probability diagnostics, predictive-entropy analysis, and frozen transfer evaluation for mangrove taxon mapping. It achieved the highest mean OA of 0.9720 and macro-F1 of 0.9406 among six models, with comparatively high performance under stricter spatial separation. Temperature scaling reduced ECE and NLL in held-out evaluation diagnostics, while predictive entropy ranked cross-validated source-domain errors with an AUROC of 0.8808. The frozen ensemble retained an OA of 0.9450 in both same-site temporal evaluations and achieved an OA of 0.7118 in four-category closed-set geographic transfer to Gaoqiao. The workflow connects taxon discrimination with explicit characterization of probability behavior and performance retention under specified deployment conditions and within reference-supported source and geographic label spaces. Additional regions and repeated acquisitions are needed to assess broader geographic portability and operational use.

## Data Availability

The datasets presented in this article are not readily available because part of the complete field- and UAV-derived reference dataset was jointly collected and curated with collaborating research teams and remains subject to data-custodian approval and ongoing research use. Access to the controlled reference data may be granted upon request, subject to approval by the relevant data custodians and applicable data-use and non-redistribution conditions. A public research package containing the core source code, trained model artifacts, the processed 2024 source-domain Sentinel-1/2 raster and mangrove mask, and supporting reproducibility materials is available at https://github.com/lgh-hnsfdx/IRSpec1D. Requests to access the datasets should be directed to Shulei Wu, wsl@hainnu.edu.cn.
