# Supplementary material for: Mangrove taxon mapping from Sentinel-1/2 using a compact one-dimensional network with probability calibration, predictive entropy, and frozen transfer evaluation
Source: Front Plant Sci. 2026 Oct 16;17:1967679. doi: 10.3389/fpls.2026.1967679 (PMC13719328; doi:10.3389/fpls.2026.1967679)
Supplement: Supplementary file 1 [file DataSheet1.pdf]

Supplementary Material

This Supplementary Material reports the complete acquisition metadata, fixed configurations, sensitivity analyses, cluster-aware uncertainty summaries, architecture, notation, algorithm, computational profile, statistical-validation framework, and data-use protocol supporting the revised manuscript.

Contents

Item	Content
Tables S1–S3	Scene-level acquisition metadata, mapping-footprint sensitivity, and the computational environment and fixed model configurations.
Tables S4–S6	Global and optical-only feature-order controls, modality-fusion control, and quantitative predictive-entropy analysis.
Tables S7–S10	Fold/ROI integrity audit, residual spatial autocorrelation, buffered spatial separation, and the matched 50-m model comparison.
Tables S11A–S11B and Algorithm S1	Layer-by-layer architecture, unified notation, and complete fold-wise training, validation-loss monitoring, early stopping, checkpoint selection, separate calibration-set temperature-scaling parameter fitting, held-out evaluation, and frozen-inference pseudocode.
Tables S12–S13	Matched model complexity and the operational profile of the deployed five-model–five-perturbation workflow.
Tables S14–S18	Seed and partition sensitivity, paired spatial-block uncertainty, and Gaoqiao plot-cluster and plot-level diagnostics.
Tables S19–S20	Statistical and spatial validation framework, and the data-use and parameter-update protocol for source-domain evaluation and frozen temporal/geographic transfer.
Table S21	Mean signed and absolute source-standardized shifts of all 14 optical–SAR features across the four shared categories in the geographic comparison.

Supplementary Tables

Supplementary Table S1. Scene-level Sentinel metadata and acquisition conditions for every experimental domain.

Domain / sensor	Final scene-level metadata and acquisition conditions
Dongzhai Harbor 2022 / Sentinel-2	Acquisition: 2022-04-07T03:05:39.024000Z; product: `S2B_MSIL2A_20220407T030539_N0510_R075_T49QDC_20240604T155105.SAFE`; S2MSI2A; tile 49QDC; absolute/relative orbit 26556/75; processing baseline 05.10; catalog cloud cover 7.941131%; TPXO10 −0.1034 m (rising); ERA5 rain 24 h 0.0000 mm, 48 h 0.1906 mm; Sentinel-1/Sentinel-2 sensing-start offset 31.594270 h.
Dongzhai Harbor 2022 / Sentinel-1	Acquisition: 2022-04-08T10:41:18.397001Z; product: `S1A_IW_GRDH_1SDV_20220408T104118_20220408T104147_042681_0517AF_8C26.SAFE`; IW_GRDH_1S; ascending; absolute/relative orbit 42681/84; VV+VH; incidence 30.9979–31.8409°; TPXO10 0.1132 m (falling); ERA5 rain 24 h 0.0000 mm, 48 h 0.0000 mm; offset 31.594270 h.
Dongzhai Harbor 2024 / Sentinel-2	Acquisition: 2024-04-01T03:05:21.024000Z; product: `S2A_MSIL2A_20240401T030521_N0510_R075_T49QDC_20240401T080849.SAFE`; S2MSI2A; tile 49QDC; absolute/relative orbit 45832/75; processing baseline 05.10; catalog cloud cover 6.113261%; TPXO10 −0.2803 m (rising); ERA5 rain 24 h 0.1000 mm, 48 h 0.2000 mm; offset 31.735922 h.
Dongzhai Harbor 2024 / Sentinel-1	Acquisition: 2024-04-02T10:49:30.343462Z; product: `S1A_IW_GRDH_1SDV_20240402T104930_20240402T104959_053254_06744F_C261.SAFE`; IW_GRDH_1S; ascending; absolute/relative orbit 53254/157; VV+VH; incidence 44.0423–44.6673° (area-weighted mean 44.3560°, center 44.3796°); TPXO10 0.2402 m (rising); ERA5 rain 24 h 0.0000 mm, 48 h 0.0000 mm; offset 31.735922 h.
Dongzhai Harbor 2026 / Sentinel-2	Acquisition: 2026-04-11T03:05:41.025000Z; product: `S2C_MSIL2A_20260411T030541_N0512_R075_T49QDC_20260411T065258.SAFE`; S2MSI2A; tile 49QDC; absolute/relative orbit 8339/75; processing baseline 05.12; catalog cloud cover 0.022037%; TPXO10 −0.4965 m (rising); ERA5 rain 24 h 0.0000 mm, 48 h 0.0000 mm; offset 7.591665 h.
Dongzhai Harbor 2026 / Sentinel-1	Acquisition: 2026-04-11T10:41:11.019782Z; product: `S1A_IW_GRDH_1SDV_20260411T104111_20260411T104140_064031_080E6A_775F.SAFE`; IW_GRDH_1S; ascending; absolute/relative orbit 64031/84; VV+VH; incidence 30.9983–31.8412°; TPXO10 0.1353 m (rising); ERA5 rain 24 h 0.0000 mm, 48 h 0.0000 mm; offset 7.591665 h.
Gaoqiao 2024 / Sentinel-2	Acquisition: 2024-04-01T03:05:21.024000Z; product: `S2A_MSIL2A_20240401T030521_N0510_R075_T49QCD_20240401T080849.SAFE`; S2MSI2A; tile 49QCD; absolute/relative orbit 45832/75; processing baseline 05.10; catalog cloud cover 2.979018%; TPXO10 −1.1142 m (rising); ERA5 rain 24 h 0.0000 mm, 48 h 0.0000 mm; offset 31.750923 h.
Gaoqiao 2024 / Sentinel-1	Acquisition: 2024-04-02T10:50:24.347967Z; product: `S1A_IW_GRDH_1SDV_20240402T105024_20240402T105049_053254_06744F_E9ED.SAFE`; IW_GRDH_1S; ascending; absolute/relative orbit 53254/157; VV+VH; product-specific incidence 41.4387–41.8873°; TPXO10 0.0465 m (rising); ERA5 rain 24 h 0.0000 mm, 48 h 0.0000 mm; offset 31.750923 h.

Note for Table S1. Product-specific Sentinel-1 incidence statistics were derived from SAFE annotation metadata. TPXO10-atlas-v2 modeled tidal elevation and ERA5 reanalysis rainfall describe acquisition conditions only; neither was used as an IR-Spec1D predictor.

Supplementary Table S2. Boundary-influence sensitivity of the four final taxon-mapping footprints.

Domain–year	Valid classified pixels, original / −10 m / −20 m	Dominant mapped taxon, original → −20 m	Largest absolute proportional change at −20 m
Dongzhai Harbor 2022	62,166 / 54,176 / 47,360	*R. stylosa* → *R. stylosa*	4.85 percentage points (*C. tagal*)
Dongzhai Harbor 2024	68,046 / 59,899 / 53,175	*R. stylosa* → *R. stylosa*	6.60 percentage points (*A. marina*)
Dongzhai Harbor 2026	60,704 / 52,277 / 45,226	*R. stylosa* → *R. stylosa*	3.31 percentage points (*A. corniculatum*)
Gaoqiao 2024	63,001 / 51,921 / 42,842	*A. marina* → *A. marina*	3.37 percentage points (*Bruguiera* complex)

Note for Table S2. Counts refer to pixels within the final mapping footprint that had valid taxon predictions. Inward erosions were applied to existing outputs without retraining, recalibration, or target-domain updating. This is a taxon-map boundary-robustness check, not an independent mangrove-extent accuracy assessment.

Supplementary Table S3. Original and revision computational environments, revision-stage profiling measurements, and fixed benchmark configurations.

(A) Experimental environments and timing

Item	Reported value
**Original experimental environment**	Experiments completed before revision: Windows 10 Professional; NVIDIA GeForce RTX 4090; 32 GB RAM; Python 3.10; PyTorch 2.0.1 with CUDA 11.8; scikit-learn 1.3.0; NumPy 1.24.3
**Revision analysis/profiling environment**	Additional sensitivity analyses, reproducibility checks, and computational profiling: Windows 11; Intel Core i7-13700KF; NVIDIA GeForce RTX 4090; Python 3.13.5; PyTorch 2.10.0+cu126; CUDA 12.6; cuDNN 9.10.2 (PyTorch-reported version code 91002)
**Applicability of profiling values**	All reported training-time, throughput, latency, and memory measurements refer to the revision profiling environment
**Revision numerical precision**	FP32; no mixed-precision training
**Revision reproducibility controls**	Default revision-stage random seed = 42 unless otherwise specified for an analysis; for neural-model runs, `cudnn.deterministic=True`; `cudnn.benchmark=False`.
**Revision five-fold model-fit time**	115.19 s total; 23.04 s/fold mean; 16.95 s median; 16.11–38.75 s range
**Revision post-hoc calibration/evaluation**	1.01 s total; timed separately and excluded from model-fit time
**Fold-specific retuning**	None; each classifier used one fixed configuration across all five outer folds; fold results were not used to modify configurations
**Model-hyperparameter search / budget**	No search or optimization before or during the formal benchmark; no search space or search budget applies. Neural internal validation supports validation-loss monitoring, early stopping, and checkpoint selection only
**Spatial-group K selection (separate from model tuning)**	$K \in \{10, 20, 30, 50, 80, 120, 200\}$; 2,000 candidate `GroupShuffleSplit` partitions per K (split seeds 0–1999), screened for category coverage and balanced proportions in an approximately 70/30 holdout; mean validation macro-F1 across five fixed FastCNN initialization seeds (42, 43, 44, 45, 46) supplied the Kneedle-inspired criterion; RF checked consistency but did not select $K = 80$
**K-selection FastCNN proxy**	It comprised a 1D convolution with 16 output channels (kernel size 3, padding 1), ReLU activation, MaxPool1D with kernel size 2, a second 1D convolution with 32 output channels (kernel size 3, padding 1), ReLU activation, adaptive global average pooling, flattening, and a linear classification layer.
**K-selection RF consistency check**	`RandomForestClassifier(random_state=42, n_jobs=-1)`; all other settings at scikit-learn defaults; same candidate-specific holdouts; auxiliary consistency check only, not used to select K^*
**Grouping and formal outer-fold assignment**	K-means: `random_state=42`, `n_init=10`; formal outer assignment: `StratifiedGroupKFold(n_splits=5, shuffle=False)`, with no random-state argument; fixed cyclic 2/1/1/1 training/validation/calibration/evaluation roles

(B) Fixed model configurations

Model / setting	Fixed configuration
**SVM**	$C = 1$; kernel=polynomial; degree=4; $\gamma =$ `scale`; `coef0`=0; `class_weight`=balanced; `probability=True`
**KNN**	`n_neighbors`=8; `weights`=uniform; `algorithm`=auto; `leaf_size`=100; metric=Minkowski; $p = 1$
**Random Forest**	100 trees; Gini; `max_depth`=5; `min_samples_split`=3; `min_samples_leaf`=3; `max_features`=sqrt; `bootstrap=True`; `class_weight`=balanced; `random_state`=42; identical configuration for the formal benchmark and 50-m buffered comparison
**1D-ConvNeXt**	dimensions=(8,16,32); depths=(2,2,2); depthwise kernel size=7; pointwise expansion ratio=4; GELU activation; LayerNorm; layer-scale initialization=10^{-6}; global average pooling followed by a linear classification head
**Lightweight 1D-ViT**	patch size=2, producing 7 input patches from the 14-feature vector; embedding dimension=32; attention heads=2; transformer layers=1; MLP hidden dimension=64; dropout=0.15; GELU activation; learnable class token; learnable positional embedding; LayerNorm; linear classification head
**IR-Spec1D**	Spectral-Domain Dropout=0.15; classifier dropout=0.36; inverted-residual blocks (expansion, channels, stride)=(1,32,1), (4,64,2), (4,64,1), (4,128,2), (4,128,1)
**Common deep-model controls**	class-weighted cross-entropy loss; AdamW optimizer; learning rate=8×10^{-4}; weight decay=10^{-3}; batch size=64; maximum epochs=200; early-stopping patience=25; label smoothing=0.01; Gaussian training noise SD=0.05 after standardization; `CosineAnnealingLR` with `T_max`=50 and `eta_min`=1×10^{-6}; internal-validation loss used only for monitoring, early stopping, and selection of the minimum-validation-loss checkpoint; prediction/inference batch size=4,096

Supplementary Table S4. Global input-order sensitivity under the matched fixed-fold design.

Matched arrangement relative to original	ΔOA	ΔMacro-F1
VV/VH block moved before Sentinel-2 sequence	+0.0076	+0.0030
One fixed random permutation of all 14 features	+0.0022	−0.0004

Note for Table S4. The original arrangement was the 12 Sentinel-2 bands followed by VV and VH. Values are differences from the matched original-order rerun, averaged across the same five spatial folds. The primary benchmark point estimates are reported separately in the main manuscript.

Supplementary Table S5. Targeted optical-only order and modality-fusion sensitivity.

Comparison relative to matched input-level original	Mean fold ΔOA / ΔMacro-F1	Pooled ΔOA	95% spatial-block CI	Pooled ΔMacro-F1 (95% CI)
Reverse Sentinel-2 order; VV/VH fixed	$-0.0018/-0.0034$	-0.0018	-0.0075 to $+0.0035$	-0.0059 (-0.0294 to $+0.0075$)
Fixed random Sentinel-2 order; VV/VH fixed	$-0.0002/+0.0013$	-0.0003	-0.0090 to $+0.0074$	$+0.0003$ (-0.0339 to $+0.0249$)
Approximately parameter-matched modality-specific dual branch	—	-0.0039	-0.0098 to $+0.0012$	-0.0011 (-0.0140 to $+0.0102$)

Note for Table S5. Differences are relative to the original input-level configuration rerun under the corresponding matched conditions. The dual-branch alternative contained 236,757 trainable parameters versus 236,309 for IR-Spec1D (448 parameters; 0.19% difference). These controls assess the tested arrangements only; they do not establish order invariance or physical optimality.

Supplementary Table S6. Quantitative analysis of predictive entropy derived from temperature-scaled held-out evaluation probabilities.

Quantity	Point estimate	95% spatial-block cluster-bootstrap CI
Misclassified / correct observations	92 / 3,235	—
Mean entropy, misclassified / correct	0.5861 / 0.0964 nats	—
Error-minus-correct mean-entropy difference	0.4897 nats	0.3066–0.7457
Entropy AUROC for ranking cross-validated errors	0.8808	0.7657–0.9499
Entropy AUPRC for ranking cross-validated errors	0.3884	0.1582–0.5839
cross-validated error prevalence	0.0277	—
Errors captured by highest entropy quartile	88.04%	—
Q4 / lower-75% error-rate ratio	22.08	7.52–92.53
Entropy vs. boundary distance, Spearman ρ	0.0064	-0.1624–0.1706
Error rate at 0–20 m / > 50 m boundary distance	11.81% / 1.58%	—
Boundary error-rate ratio (0–20 m / > 50 m)	7.50	1.60–31.42
Entropy vs. Sentinel-2 local heterogeneity, Spearman ρ	0.0732	-0.0690–0.2097
Heterogeneity Q4–Q1 mean-entropy difference	0.0982 nats	0.0418–0.1595
Error rate in heterogeneity Q1 / Q4	0.48% / 8.89%	—

Note for Table S6. Probabilities were pooled from the held-out evaluation sets $\mathscr{D}_f^{\text{eval}}$, using checkpoints selected on the internal validation subsets $\mathscr{D}_f^{\text{val}}$ and temperature-scaling parameters fitted only on the separate calibration subsets $\mathscr{D}_f^{\text{cal}}$. Intervals use 5,000 bootstrap replicates of the 80 fixed spatial blocks. Boundary distance was measured from the final 2024 binary mangrove mask; local Sentinel-2 heterogeneity used the prespecified 3×3-pixel procedure described in the Methods.

Supplementary Table S7. Outer-fold integrity and nearest-training separation under the primary protocol.

Evaluation fold	Samples	Spatial groups	Minimum distance (m)	Median distance (m)
1	677	17	10	30.0
2	676	16	10	31.6
3	678	16	10	28.3
4	636	15	10	33.8
5	660	16	10	30.0

Note for Table S7. The 373 ROI units were the original field/UAV-informed reference delineations established before fold assignment. A manual integrity audit used these original units and confirmed that each was assigned wholly to one fold (373/373; 0 split). All 80 coordinate-defined spatial groups were likewise intact (0/80 split), and no identical reference-pixel coordinate occurred in different folds.

Supplementary Table S8. Moran's *I* sensitivity for the pooled cross-validated binary classification-error indicator.

Spatial-weight definition	Parameter	Moran's *I*	Permutation *p*
k-nearest neighbor	$k = 4$	0.562	< 0.001
k-nearest neighbor (primary)	$k = 8$	0.4968	< 0.001
k-nearest neighbor	$k = 12$	0.459	< 0.001
Distance band	20 m	0.504	< 0.001
Distance band	30 m	0.434	< 0.001
Distance band	50 m	0.335	< 0.001

Note for Table S8. Weights were row-standardized and significance was assessed with 9,999 permutations. The primary 8-nearest-neighbor value is reported to four decimal places in the manuscript; the alternative values are rounded to three decimals. Positive values indicate residual spatial clustering of errors and do not establish leakage or complete spatial independence.

Supplementary Table S9. Buffered spatial-separation sensitivity of IR-Spec1D.

Nominal exclusion radius	Actual minimum separation (m)	Mean training observations excluded (%)	Mean fold OA ± SD	Mean fold macro-F1 ± SD	Pooled OA / macro-F1
20 m	22.36	10.7	0.9591 ± 0.0197	0.8809 ± 0.0763	0.9594 / 0.9096
50 m	50.99	30.9	0.9443 ± 0.0321	0.8587 ± 0.1010	0.9441 / 0.8949
100 m	100.50	51.4	0.9317 ± 0.0353	0.8626 ± 0.0686	0.9318 / 0.8929

Note for Table S9. Outer evaluation folds were unchanged. Training observations within each nominal radius of any held-out evaluation observation were excluded before model fitting. Buffering jointly increases spatial separation and reduces training-set size, so performance changes cannot be attributed to either factor alone.

Supplementary Table S10. Matched model comparison under the 50 m exclusion buffer.

Model	Mean fold OA ± SD	Mean fold macro-F1 ± SD	Pooled OA	Pooled macro-F1
IR-Spec1D	0.9443 ± 0.0321	0.8587 ± 0.1010	0.9441	0.8949
KNN	0.9398 ± 0.0268	0.8469 ± 0.0860	0.9399	0.8751
RF	0.9165 ± 0.0350	0.8572 ± 0.0777	0.9161	0.8891
1D-ConvNeXt	0.9124 ± 0.0414	0.7747 ± 0.0805	0.9122	0.8015

Note for Table S10. All models used the same fixed outer evaluation folds, the same retained training observations, and the same preprocessing and within-fold training/internal-validation roles. IR-Spec1D had the highest point estimates in this descriptive matched comparison; no claim of statistical superiority is made.

Supplementary Table S11A. Layer-by-layer architecture of IR-Spec1D.

Stage	Input ($C \times L$)	Operation and component position	Expansion t	Kernel / stride / padding	Activation	Residual condition	Output ($C \times L$)	Parameters
Input	1×14	Standardized Sentinel-2/Sentinel-1 feature vector	—	—	—	—	1×14	0
Spectral-Domain Dropout	1×14	Input feature masking before the stem ($p = 0.15$; training only)	—	—	—	—	1×14	0
Stem	1×14	Conv1D $1 \rightarrow 32$; BN	—	$k = 3, s = 1, p = 1$	SiLU after BN	No	32×14	160
IR block 1	32×14	DWConv1D $32 \rightarrow 32$; BN; PW projection $32 \rightarrow 32$; BN; ECA after projection	1	DW: $k = 3, s = 1, p = 1$; PW: $k = 1, s = 1, p = 0$; ECA: $k = 3, p = 1$	SiLU after DW; none after projection; ECA sigmoid gating	Yes: $s = 1$, channels match	32×14	1,251
IR block 2	32×14	PW expansion $32 \rightarrow 128$; BN; DWConv1D $128 \rightarrow 128$; BN; PW projection $128 \rightarrow 64$; BN; ECA after projection	4	PW: $k = 1, s = 1, p = 0$; DW: $k = 3, s = 2, p = 1$; ECA: $k = 3, p = 1$	SiLU after expansion and DW; none after projection; ECA sigmoid gating	No: $s = 2$, channels differ	64×7	13,315
IR block 3	64×7	PW expansion $64 \rightarrow 256$; BN; DWConv1D $256 \rightarrow 256$; BN; PW projection $256 \rightarrow 64$; BN; ECA after projection	4	PW: $k = 1, s = 1, p = 0$; DW: $k = 3, s = 1, p = 1$; ECA: $k = 3, p = 1$	SiLU after expansion and DW; none after projection; ECA sigmoid gating	Yes: $s = 1$, channels match	64×7	34,691
IR block 4	64×7	PW expansion $64 \rightarrow 256$; BN; DWConv1D $256 \rightarrow 256$; BN; PW projection $256 \rightarrow 128$; BN; ECA after projection	4	PW: $k = 1, s = 1, p = 0$; DW: $k = 3, s = 2, p = 1$; ECA: $k = 3, p = 1$	SiLU after expansion and DW; none after projection; ECA sigmoid gating	No: $s = 2$, channels differ	128×4	51,203
IR block 5	128×4	PW expansion $128 \rightarrow 512$; BN; DWConv1D $512 \rightarrow 512$; BN; PW projection $512 \rightarrow 128$; BN; ECA after projection	4	PW: $k = 1, s = 1, p = 0$; DW: $k = 3, s = 1, p = 1$; ECA: $k = 3, p = 1$	SiLU after expansion and DW; none after projection; ECA sigmoid gating	Yes: $s = 1$, channels match	128×4	134,915
Classification head	128×4	Adaptive GAP $\rightarrow$ Flatten $\rightarrow$ Dropout ($p = 0.36$; training only) $\rightarrow$ Linear $128 \rightarrow 6$	—	—	None after linear	No	6 logits	774
Total	—	Complete six-category IR-Spec1D	—	—	—	—	6 logits	236,309

Note for Table S11A. Within Table S11A, C denotes channel dimension and L denotes feature-axis length; the category count is defined separately in Table S11B. PW denotes pointwise convolution; DWConv1D denotes depthwise one-dimensional convolution; BN denotes batch normalization; ECA denotes Efficient Channel Attention. The table reports dropout at its point of application and gives residual eligibility for each block directly.

Supplementary Table S11B. Notation and symbols used in the IR-Spec1D workflow.

Symbol	Definition	Role / dimension	Unit, range, or experimental value
r	Pixel/sample index	Reference sample or mapped pixel	Positive integer index; no physical unit
f	Outer-fold model index	$f = 1,\dots,F$	Integer; $f = 1,\dots,5$
c	Classification-category index	$c = 1,\dots,C$	Integer; $c = 1,\dots,6$ in source classification
m	TTA pass index	$m = 1,\dots,M$	Integer; $m = 1,\dots,5$ for scene/transfer inference
K	Number of coordinate-defined spatial groups	80	Integer; $K = 80$ in the formal grouping
F	Number of outer folds/models	5	Integer; $F = 5$
C	Number of source classification categories	6	Gaoqiao uses four prespecified shared categories after restriction and renormalization
M	Number of scene/transfer TTA realizations	5	One unperturbed plus four Gaussian-perturbed passes
$\mathscr{D}_{\mathrm{src}}$	Source-domain reference dataset	All source observations before fold-specific roles	Dataset; no physical unit
$\mathscr{D}_f^{\mathrm{tr}}$	Fold-specific source training subset: two folds	StandardScaler fitting and gradient-based network optimization	Fixed cyclic allocation; separate from the other three roles
$\mathscr{D}_f^{\mathrm{val}}$	Fold-specific internal validation subset: one fold	Validation-loss monitoring, early stopping, and checkpoint selection under fixed model settings	No architecture or model-hyperparameter selection; separate from training, calibration, and held-out evaluation
$\mathscr{D}_f^{\mathrm{cal}}$	Fold-specific calibration subset: one fold	Temperature-scaling parameter fitting only, after restoring the selected checkpoint	No network fitting or early stopping; separate from the other three roles
$\mathscr{D}_f^{\mathrm{eval}}$	Held-out outer evaluation subset: one fold	Source-domain classification and probability metrics	Excluded from scaler/model fitting, early stopping, checkpoint selection, configuration changes, and temperature-scaling parameter fitting after K was fixed
$N_f^{\mathrm{tr}}, N_{f,c}^{\mathrm{tr}}$	Training-set size and category-specific training support	Scalar counts; used to compute $w_{f,c}$	Positive integers; samples
N_f^{val}	Internal-validation subset size	Scalar count; internal validation only	Positive integer; samples
N_f^{cal}	Calibration-subset size	Scalar count in the temperature-scaling objective	Positive integer; samples
N_f^{eval}	Held-out evaluation-fold size	Scalar count in ECE and NLL	Positive integer; samples
$\mathbf{x}_r$	Original 14-dimensional Sentinel-2/Sentinel-1 vector	$\mathbb{R}^{14}$	12 optical surface-reflectance components (dimensionless) and two SAR backscatter components (dB)
$\mu_f, \mathbf{s}_f$	Feature-wise mean and scale fitted from $\mathscr{D}_f^{\mathrm{tr}}$	$\mathbb{R}^{14}$ each	Feature-specific units before standardization
$\widetilde{\mathbf{x}}_{r,f}$	Fold-standardized vector	$\mathbb{R}^{14}$	Dimensionless; 14 components
θ_f	Learned parameters of fold model f	IR-Spec1D parameters	Learned numerical quantities
$g_{\theta_f}(\cdot)$	Complete trained IR-Spec1D mapping	$\mathbb{R}^{14} \to \mathbb{R}^{C}$	Function; no physical unit
$\mathbf{z}_{r,f}$	Source-category logit vector	$\mathbb{R}^{C}$	Dimensionless; six logits
$\mathbf{z}_{n,f}^{\mathrm{cal}}$	Logits for calibration sample n from the retained fold checkpoint	$\mathbb{R}^{C}$	Dimensionless; six logits
T_f	Positive post-hoc temperature-scaling parameter estimated from $\mathscr{D}_f^{\mathrm{cal}}$ logits after network training and checkpoint selection	Scalar	Dimensionless; $T_f > 0$; fixed before held-out evaluation and scene/transfer inference
$\mathscr{L}_{\mathrm{cal},f}(T)$	Regularized post-hoc temperature-scaling objective on $\mathscr{D}_f^{\mathrm{cal}}$	Scalar loss; fitted after checkpoint selection	Dimensionless; $\lambda = 10^{-3}$
$y_{n,c}$	One-hot reference label for sample n and category c	Scalar component of a C-category label vector	$y_{n,c} \in \{0,1\}$; $\sum_c y_{n,c} = 1$
$b, B_{f,b}$	Confidence-bin index and set of held-out evaluation samples in that bin	Index and subset used in ECE	$b = 1,\dots,15$; equal-width bins over $[0,1]$
$\mathscr{S}$	Prespecified shared-category set for Gaoqiao	Subset of the source label space	Four shared categories
$\xi_{r,f}^{(m)}$	Gaussian TTA perturbation	$\mathbb{R}^{14}$	Standardized-feature units; SD=0.05 for $m = 2,\dots,5$; $m = 1$ is zero
$\pi_{r,f,c}^{(m)}$	Temperature-scaled pass probability	$[0,1]$	Class probabilities sum to one
$q_{r,f,c}$	Within-fold probability averaged over TTA passes	$[0,1]$	Class probabilities sum to one
$\overline{\pi}_{r,c}$	Final ensemble probability	$[0,1]$	$\sum_c \overline{\pi}_{r,c} = 1$
$\widehat{y}_r$	Final predicted category	$\arg\max_c \overline{\pi}_{r,c}$	Discrete category label
H_r	Predictive entropy	Scalar	Nats; $0 \le H \le \ln C$
$\varepsilon_{\mathrm{num}}$	Numerical constant preventing $\log(0)$	Scalar	Dimensionless; 10^{-12}
$\mathbf{H}$	Intermediate feature tensor entering ECA	Channels $\times$ feature-axis length	Learned activations; block-dependent shape
ω	ECA channel-attention weights from GAP($\mathbf{H}$)	Channel-wise weights	Dimensionless sigmoid output in $[0,1]$
$\mathbf{H}_{r,f}^{\mathrm{out}}$	Final ECA-reweighted feature tensor	128×4 before GAP	Learned feature activations
$\mathbf{W}_f, \mathbf{b}_f$	Final linear-layer weight matrix and bias	$\mathbb{R}^{6\times 128}$ and $\mathbb{R}^{6}$	Learned numerical quantities
$\mathscr{D}$	Dropout operator in the classification head	Dropout probability 0.36 in training	Disabled during evaluation/inference
$w_{f,c}$	Training loss weight for category c in fold f	Class-balanced scalar	Positive, dimensionless, fold/category specific
λ	Temperature-scaling regularization coefficient	Scalar	Dimensionless; 10^{-3}

Supplementary Algorithm

Algorithm S1. Fold-specific IR-Spec1D training, calibration, and ensemble inference

Input Source-domain reference dataset $\mathscr{D}_{\text{src}}$ with 14 features; fixed $K = 80$ spatial groups; $F = 5$ outer folds; $C = 6$ source categories.

Output Fold models $\{g_{\theta_f}\}$, fold scalers $\{(\mu_f, \mathbf{s}_f)\}$, fitted temperature-scaling parameters $\{T_f\}$, ensemble probabilities, predicted category, and predictive entropy.

K-means grouping used `random_state=42` and `n_init=10`. Formal outer-fold assignment used `StratifiedGroupKFold` with `n_splits=5` and `shuffle=False`; no random-state argument was supplied. Each classifier used one fixed configuration across all five outer folds; no model-hyperparameter search or optimization was performed. The cyclic allocation below uses intact folds, not fixed sample percentages.

Outer iteration	$\mathscr{D}_f^{\text{tr}}$	$\mathscr{D}_f^{\text{val}}$	$\mathscr{D}_f^{\text{cal}}$	$\mathscr{D}_f^{\text{eval}}$
1	Folds 4 + 5	Fold 2	Fold 3	Fold 1
2	Folds 5 + 1	Fold 3	Fold 4	Fold 2
3	Folds 1 + 2	Fold 4	Fold 5	Fold 3
4	Folds 2 + 3	Fold 5	Fold 1	Fold 4
5	Folds 3 + 4	Fold 1	Fold 2	Fold 5

1. For each outer fold $f = 1, \ldots, F$:
2. Assign fold f to $\mathscr{D}_f^{\text{eval}}$, the next cyclic fold to $\mathscr{D}_f^{\text{val}}$, the following fold to $\mathscr{D}_f^{\text{cal}}$, and the remaining two to $\mathscr{D}_f^{\text{tr}}$, as shown above.
3. Fit StandardScaler on $\mathscr{D}_f^{\text{tr}}$ only to obtain μ_f and $\mathbf{s}_f$.
4. Apply the same transformation to $\mathscr{D}_f^{\text{val}}$, $\mathscr{D}_f^{\text{cal}}$, and $\mathscr{D}_f^{\text{eval}}$ and later scene/transfer inputs without refitting.
5. Initialize the six-category IR-Spec1D model g_{θ_f}.
6. During training, apply Spectral-Domain Dropout with $p = 0.15$ and Gaussian feature noise with SD=0.05 after standardization.
7. On $\mathscr{D}_f^{\text{tr}}$ only, optimize class-weighted cross-entropy with label smoothing 0.01 using AdamW (learning rate 8×10^{-4}, weight decay 10^{-3}) and cosine annealing.
8. Under the fixed configuration, train with batch size 64 for at most 200 epochs. Monitor loss on $\mathscr{D}_f^{\text{val}}$ with dropout and training perturbations disabled; stop after 25 epochs without improvement and restore the minimum-loss checkpoint. Do not use $\mathscr{D}_f^{\text{val}}$ to select the architecture or model hyperparameters.
9. Freeze the retained network parameters θ_f.
10. With dropout and training perturbations disabled, compute logits on $\mathscr{D}_f^{\text{cal}}$ with the retained checkpoint and fit positive T_f to these logits and labels by minimizing regularized NLL with $\lambda = 10^{-3}$ using L-BFGS. Fix T_f before held-out evaluation.
11. With dropout disabled, compute OA, macro-F1, and probability diagnostics on $\mathscr{D}_f^{\text{eval}}$ using one unperturbed input per sample, without TTA or five-model averaging. Calculate ECE/NLL before and after temperature scaling; positive scalar scaling preserves the categorical argmax.
12. Save the fold-specific scaler, retained checkpoint, and T_f; keep them frozen for scene-level and transfer inference.
13. End fold loop.
14. For scene-level or transfer inference at pixel r:
15. For each fold model f, standardize $\mathbf{x}_r$ with $(\mu_f, \mathbf{s}_f)$.
16. Generate $M = 5$ inputs: one unperturbed vector and four independent Gaussian perturbations with SD=0.05 in standardized feature space.
17. Disable activation dropout and Spectral-Domain Dropout; compute logits for every pass.
18. Apply temperature scaling and Softmax to every pass-level logit vector.
19. Average pass-level probabilities within each fold to obtain $q_{r,f,c}$.
20. Average $q_{r,f,c}$ across the five fold models to obtain $\overline{\pi}_{r,c}$.
21. For Gaoqiao closed-set transfer, restrict $\overline{\pi}_{r,c}$ to the prespecified shared categories and renormalize it; in the two following steps, $\overline{\pi}_{r,c}$ denotes this resulting four-category vector.
22. Predict $\widehat{y}_r = \arg\max_c \overline{\pi}_{r,c}$.
23. Compute $H_r = -\sum_c \overline{\pi}_{r,c} \log(\overline{\pi}_{r,c} + \varepsilon_{\text{num}})$.
24. Return the final probability vector, predicted category, and predictive entropy.

Supplementary Table S12. Matched model complexity and CPU/GPU inference profile.

Model	Parameters	FP32 state dictionary (MB)	MFLOPs/sample	CPU throughput, batch 4096 (samples/s)	RTX 4090 throughput, batch 4096 (samples/s)	Peak GPU memory (MB)
IR-Spec1D	236,309	0.9530	2.4393	34,384	486,956	93.98
1D-ConvNeXt	24,326	0.1146	0.7530	101,345	1,758,969	94.15
Lightweight 1D-ViT	9,190	0.0415	0.0712	753,664	6,083,921	38.46

Note for Table S12. These revision-stage measurements were obtained in the Windows 11 profiling environment specified in Table S3. Batch-1 mean latencies (CPU / RTX 4090) were 2.6849 / 1.3557 ms for IR-Spec1D, 1.8977 / 1.2297 ms for 1D-ConvNeXt, and 0.2601 / 0.3947 ms for Lightweight 1D-ViT. MACs were measured with THOP; FLOPs $= 2\times$ MACs. All values use the matched profiling procedure described in the manuscript.

Supplementary Table S13. Direct operational profile of the deployed IR-Spec1D ensemble.

Workflow	Device	Throughput (valid pixels/s)	Seconds per one million valid pixels	Seconds per km^2 at 10 m	Peak GPU memory (MB)
Five models $\times$ five perturbations (25 passes)	CPU	1,200.57	832.94	8.329	—
Five models $\times$ five perturbations (25 passes)	RTX 4090	15,001.72	66.659	0.6666	93.82

Note for Table S13. These revision-stage measurements were obtained in the Windows 11 profiling environment specified in Table S3. Timing includes fold-specific StandardScaler transformation, host-to-device transfer, Gaussian TTA, temperature scaling, Softmax, five-model averaging, argmax, and predictive entropy; disk input/output and map rendering are excluded. Synthetic 14-dimensional inputs were used only for throughput timing; no accuracy metrics were computed during profiling.

Supplementary Table S14. Training-stochasticity sensitivity of IR-Spec1D under five prespecified random seeds and fixed spatial partitions.

Seed	Mean OA across five fixed folds	Fold SD for OA	Mean macro-F1 across five fixed folds	Fold SD for macro-F1
42	0.9720	0.0188	0.9406	0.0456
43	0.9724	0.0154	0.9382	0.0474
44	0.9717	0.0191	0.9054	0.0666
45	0.9733	0.0137	0.9097	0.0704
46	0.9742	0.0142	0.9487	0.0382
Mean $\pm$ seed-to-seed SD	0.9727 ± 0.0010	—	0.9285 ± 0.0196	—
Minimum–maximum	0.9717–0.9742	—	0.9054–0.9487	—

Note for Table S14. Performance was first averaged across the five fixed outer folds within each seed. The summary SD is the seed-to-seed SD of the five seed-level means; within-row SD instead describes fold-to-fold variation.

Supplementary Table S15. Repeated spatial-partition sensitivity of IR-Spec1D versus KNN.

Partition seed	IR OA	IR macro-F1	KNN OA	KNN macro-F1	ΔOA	ΔMacro-F1
1	0.9700	0.9468	0.9592	0.8908	+0.0108	+0.0560
2	0.9715	0.9387	0.9592	0.8904	+0.0123	+0.0483
3	0.9739	0.9489	0.9608	0.8939	+0.0131	+0.0551
4	0.9718	0.9401	0.9613	0.8948	+0.0105	+0.0453
5	0.9745	0.9418	0.9602	0.9001	+0.0143	+0.0417
Mean $\pm$ across-partition SD	0.9723 ± 0.0019	0.9432 ± 0.0044	0.9602 ± 0.0009	0.8940 ± 0.0039	$+0.0122 \pm 0.0016$	$+0.0493 \pm 0.0062$

Note for Table S15. Five alternative group-disjoint five-fold assignments were generated from the same 80 fixed spatial groups. Architecture, preprocessing, model hyperparameters, and training seed were fixed; only group-to-fold allocation varied. KNN used the same partitions. Values in rows 1–5 are fold means within each partition. Both aggregate differences were positive in all five partitions. For pooled cross-validated predictions, the corresponding across-partition means were $\Delta OA = +0.0122 \pm 0.0015$ and $\Delta\text{Macro-F1} = +0.0524 \pm 0.0069$.

Supplementary Table S16. Paired spatial-block bootstrap uncertainty for IR-Spec1D versus KNN.

Metric	Pooled cross-validated IR-Spec1D	Pooled cross-validated KNN	Paired difference	95% bootstrap CI	Bootstrap proportion with $\Delta > 0$
OA	0.97235	0.96063	+0.01172	0.00305–0.02444	0.9993
Macro-F1	0.94407	0.89786	+0.04621	0.00317–0.11862	0.9938

Note for Table S16. The formal benchmark cross-validated predictions used training seed 42 for IR-Spec1D; KNN is deterministic for the fixed groups and folds. The 80 fixed spatial blocks were sampled with replacement for 10,000 paired replicates using resampling seed 20260829. Every replicate retained all pixels from selected blocks and used the same blocks for both models. The final column is descriptive and is not a conventional hypothesis-test p-value. Pooled cross-validated metrics differ from arithmetic means of fold-level metrics in the main manuscript.

Supplementary Table S17. Gaoqiao class-stratified plot-cluster bootstrap uncertainty.

(A) Overall frozen closed-set performance

Metric	Point estimate	95% plot-cluster CI
OA	0.7118	0.5799–0.8368
Macro-F1	0.7131	0.5754–0.8354

(B) Category-specific performance

Category	Metric	Point estimate	Bootstrap mean	95% plot-cluster CI
A. marina	Precision	0.6282	0.6350	0.5055–0.7846
	Recall	0.6806	0.6809	0.5833–0.7500
	F1	0.6533	0.6549	0.5610–0.7482
B. sexangula/*B. gymnorhiza* complex	Precision	0.7667	0.7742	0.6229–0.9333
	Recall	0.6389	0.6390	0.5000–0.7361
	F1	0.6970	0.6973	0.5846–0.8000
R. stylosa	Precision	0.5897	0.5916	0.4853–0.7000
	Recall	0.6389	0.6385	0.5135–0.7361
	F1	0.6133	0.6126	0.5098–0.7051
A. corniculatum	Precision	0.8889	0.8891	0.8235–0.9538
	Recall	0.8889	0.8880	0.7639–0.9861
	F1	0.8889	0.8875	0.8085–0.9524

Note for Table S17. Each shared category contains 72 reference pixels from eight distinct field plots. Within each category, eight plots were sampled with replacement and all nine adjacent samples from each selected plot were retained. Point estimates are the original frozen Gaoqiao closed-set metrics; bootstrap means do not replace them. Intervals are percentile 95% CIs from 10,000 class-stratified plot-cluster replicates.

Supplementary Table S18. Plot-level confusion matrix after mean-probability aggregation across the nine samples within each Gaoqiao field plot.

Reference category	Predicted *A. marina*	Predicted *Bruguiera* complex	Predicted *R. stylosa*	Predicted *A. corniculatum*
A. marina	7	0	1	0
B. sexangula/*B. gymnorhiza* complex	1	5	2	0
R. stylosa	2	1	5	0
A. corniculatum	0	0	0	8

Note for Table S18. Plot prediction was defined by averaging the four frozen class probabilities across the nine samples in each plot and selecting the largest mean probability. Of 32 plots, 25 were classified correctly, corresponding to plot-level OA = 0.7813, macro-F1 = 0.7793, and Cohen's $\kappa = 0.7083$. This diagnostic complements, rather than replaces, the sample-level point estimates and plot-cluster intervals.

Supplementary Table S19. Statistical and spatial validation framework used in the robustness analyses.

Analysis	Evaluation / resampling unit	What varied / was resampled	What remained fixed	Number of units / repetitions	Primary purpose	Detailed-results location
Primary grouped cross-validation	Fold-assignment unit: coordinate-defined spatial group; prediction unit: held-out evaluation pixel	Fixed cyclic allocation: two folds for fitting, one for validation-loss monitoring, early stopping, and checkpoint selection, one for temperature-scaling parameter fitting, and one for held-out evaluation	Fixed $K = 80$ grouping, six-category label space, 14 inputs, and model configurations; evaluation observations excluded from fitting and selection after K was fixed	3,327 observations; 80 groups; five folds	Summarize source-domain discrimination and probability diagnostics conditional on the data-informed grouping	Main manuscript Figure 5A and Table 2
ROI/group integrity audit	Reference ROI, coordinate-defined spatial group, and reference-pixel coordinate	No resampling; manual audit of original ROI memberships, supplemented by group-integrity and duplicate-coordinate checks	Primary $K = 80$ fold assignment and original field/UAV-informed reference ROI delineations	373 ROI units; 80 groups; 3,327 observations	Verify that ROI and group units were not split and that identical pixel coordinates did not occur across folds	Supplementary Table S7
Residual Moran's I	Held-out evaluation reference pixel in the pooled cross-validated binary classification-error field	Spatial-weight definition was varied; permutation assessment was used for significance	Observed pooled cross-validated predictions and projected reference coordinates; 8-nearest-neighbor weights were primary	3,327 cross-validated observations; six weight definitions; 9,999 permutations	Quantify residual spatial clustering of errors, not diagnose leakage	Supplementary Table S8
Buffer validation	Training reference observation was the exclusion unit; evaluation remained on the fixed evaluation observations and folds	Training observations within 20, 50, or 100 m of held-out evaluation observations were excluded; four models were compared at 50 m	Outer evaluation observations and folds; final configurations; matched preprocessing and training/internal-validation roles; identical retained training data in the 50-m comparison	Three radii across five folds; four models in the 50-m comparison	Test sensitivity to increasing short-range training–evaluation separation	Supplementary Tables S9–S10
Five training seeds	Training seed; results summarized as a five-fold mean for each seed	IR-Spec1D training seed varied from 42 to 46	Spatial partition, within-fold data roles, preprocessing, architecture, and hyperparameters	Five seeds; five folds per seed	Characterize training stochasticity separately from fold-to-fold variation	Supplementary Table S14
Five alternative partitions	Group-to-fold assignment; results summarized as a five-fold mean for each partition	The same 80 fixed spatial groups were reassigned using partition seeds 1–5	Spatial groups, architecture, preprocessing, hyperparameters, and training seed; KNN used the same assignments	Five assignments; five folds per assignment; two matched models	Test dependence on the particular group-to-fold allocation	Supplementary Table S15
Paired spatial-block bootstrap	Fixed spatial block, with all pixels in a sampled block retained together	The 80 blocks were sampled with replacement, using identical sampled blocks for IR-Spec1D and KNN	Formal-benchmark cross-validated predictions; IR-Spec1D seed 42; deterministic KNN predictions; paired comparison	80 blocks; 10,000 paired replicates	Quantify paired uncertainty in pooled cross-validated ΔOA and ΔMacro-F1 while preserving spatial units	Supplementary Table S16
Gaoqiao plot-cluster bootstrap	Field plot, stratified by the four shared categories	Within each category, eight plots were sampled with replacement from the eight available plots, retaining all nine adjacent samples from each selected plot	Frozen source networks, fold-specific scalers, fitted temperature-scaling parameters, and the prespecified four-category closed-set label space	32 plots; eight plots per category; nine samples per plot; 10,000 replicates	Estimate cluster-aware intervals without treating the 288 adjacent samples as independent	Supplementary Table S17
Gaoqiao plot-level aggregation	Field plot	No resampling; four frozen class probabilities were averaged across the nine samples in each plot before maximum-probability assignment	Frozen source pipeline and prespecified four-category label space; sample-level point estimates and plot-cluster intervals remained primary	32 plots; nine samples per plot	Provide a secondary plot-level confusion-matrix diagnostic rather than replace the sample-level analysis	Supplementary Table S18

Note for Table S19. This table distinguishes fold-based evaluation, deterministic integrity auditing, spatial sensitivity analyses, training and partition repetitions, and cluster-aware resampling. All supplementary model-fitting comparisons retained the cyclic 2/1/1/1 fold-role allocation, including within each alternative partition. Buffered analyses removed observations only from the assigned training folds. Classification-only controls did not refit temperature-scaling parameters and did not reassign the reserved calibration fold to training or validation. The separate approximately 70/30 proxy holdout selected K before formal cross-validation; K was not reselected within each outer iteration. The source-domain estimates remain conditional on this data-informed grouping. Numerical results remain in the cited main-manuscript and Supplementary locations.

Supplementary Table S20. Data-use and parameter-update protocol for source-domain evaluation and frozen temporal/geographic transfer.

Data subset / operation	Permitted role	Fitted, selected, or computed quantity	Explicitly fixed / excluded use
Source training subset, $\mathscr{D}_f^{\mathrm{tr}}$: two folds	Fit the fold-specific `StandardScaler` and optimize the six-category network using only the two assigned training folds	Feature means and scales $(\boldsymbol{\mu}_f, \mathbf{s}_f)$ and network parameters $\boldsymbol{\theta}_f$	Internal validation, calibration, and held-out evaluation observations excluded from scaler fitting and gradient-based optimization
Source internal validation subset, $\mathscr{D}_f^{\mathrm{val}}$: one fold	Validation-loss monitoring, early stopping, and checkpoint selection under fixed model settings	Minimum-validation-loss checkpoint; no architecture or model-hyperparameter selection	No scaler fitting, gradient-based optimization, temperature-scaling parameter fitting, or reported held-out metrics
Source calibration subset, $\mathscr{D}_f^{\mathrm{cal}}$: one fold	Fit the post-hoc temperature-scaling parameter after restoring the selected checkpoint	T_f, estimated by regularized NLL minimization	Retained network and scaler fixed; no early stopping, checkpoint selection, or reported held-out metrics
Source held-out evaluation set, $\mathscr{D}_f^{\mathrm{eval}}$: one fold	Compute source-domain classification and probability diagnostics using the corresponding fixed fold model, scaler, and temperature-scaling parameter	OA, macro-F1, ECE, NLL, and associated probabilities	After K was fixed, no fitting, early stopping, checkpoint selection, configuration changes, or temperature-scaling parameter estimation; one unperturbed pass, no TTA or five-model averaging
Dongzhai Harbor 2022/2026 temporal-target imagery and reference samples	Apply the frozen source ensemble to target imagery; use target reference labels only for numerical evaluation	Frozen-inference outputs and evaluation metrics; no fitted parameter	No model fitting, preprocessing refitting, recalibration, adaptation, or model updating; target references did not contribute to map production
Gaoqiao imagery and reference samples	Apply the frozen source pipeline in the prespecified four-category closed-set setting; use reference labels only for evaluation	Closed-set probabilities, map, predictive entropy, and evaluation metrics; no fitted parameter	No training, fine-tuning, configuration decision, `StandardScaler` fitting, temperature-scaling refitting, recalibration, adaptation, or model updating
Scene/transfer TTA	For each fold model, use one unperturbed input and four independent Gaussian perturbations with SD=0.05 in standardized feature space; inference only	Within-fold probability average $q_{r,f,c}$; no parameter update	Activation dropout and Spectral-Domain Dropout remained disabled; TTA was not used for source held-out evaluation
Five-model probability averaging	Average within-fold probabilities across the five fixed fold models; inference only	Final ensemble probability vector $\overline{\boldsymbol{\pi}}_r$; no parameter update	Network weights were not averaged; networks, scalers, and temperature-scaling parameters remained fixed; this averaging was not used for source held-out evaluation
Gaoqiao four-category restriction and renormalization	Restrict six-category ensemble probabilities to the prespecified shared-category set and renormalize them for the Gaoqiao closed-set map and numerical evaluation	Four-category probability vector $\overline{\boldsymbol{\pi}}_r^{(\mathscr{S})}$; no fitted parameter	Probability post-processing only; no training, fine-tuning, recalibration, adaptation, or open-set recognition

Note for Table S20. No target-domain reference label was used for parameter updating. The prespecified shared-category set was used to define the Gaoqiao closed-set label space. Using target-domain observations for model updating would constitute a target-adaptation experiment rather than the fully frozen transfer protocol evaluated here. Detailed execution order is reported in Supplementary Algorithm S1.

Supplementary Table S21. Mean source-standardized shifts of all 14 optical–SAR input features between Dongzhai Harbor and Gaoqiao across the four shared categories.

Feature	Mean signed shift	Mean absolute shift	Direction across four categories
B2	7.549	7.549	Positive
B1	7.526	7.526	Positive
B3	6.046	6.046	Positive
B4	4.867	4.867	Positive
B5	3.829	3.829	Positive
B9	1.779	1.779	Positive
B6	1.579	1.579	Positive
B8A	1.440	1.440	Positive
B7	1.438	1.438	Positive
B8	1.361	1.361	Positive
B11	0.662	1.143	Mixed
B12	−0.161	1.025	Mixed
VV	0.005	0.796	Mixed
VH	0.139	0.507	Mixed

Note for Table S21. The analysis used 1,749 source observations from the four shared categories and 288 Gaoqiao observations from 32 field plots. For each feature and category, the target-minus-source class-conditional mean difference was divided by that feature's standard deviation across the four shared source-domain categories. All 14 input features are reported: 12 Sentinel-2 optical bands and Sentinel-1 VV/VH. Mean signed shift is the arithmetic mean of the four category-specific standardized differences. Mean absolute shift averages their absolute values, rather than taking the absolute value of the signed mean. Positive indicates a positive difference in all four categories; Mixed indicates both positive and negative differences across categories. Values are dimensionless descriptive effect sizes in source-standard-deviation units, not significance tests or causal attributions.

Reproducibility materials

The public research package is available at `https://github.com/lgh-hnsfdx/IRSpec1D`. The repository contains the two core source-code implementations used for spatial-group K-selection and the IR-Spec1D workflow, fixed configuration information, five fold-specific checkpoints with StandardScaler objects and fitted temperature-scaling parameters, the processed 2024 Dongzhai Harbor 14-feature raster and mangrove mask, scene metadata, aggregate fold-level and spatial-audit summaries, and the released 60-example source-domain subset with its fold-specific verification outputs. The complete reference-sample table, exact coordinates and grid positions, complete sample-level feature and label records beyond the released example subset, complete sample-to-spatial-group and sample-to-fold/role manifests, and ROI-level location and partition records remain under controlled access as described in the Data Availability Statement. The public materials support independent inspection of the implementation, fixed experimental settings, archived model artifacts, Sentinel inputs, aggregate diagnostics, the K-selection protocol, and limited fold-specific held-out verification using the released example subset. Exact recomputation of the complete reference-based analyses requires authorized access to the controlled reference data.
